# Supplementary figures and images for: Leveraging functional annotation to identify genes associated with complex diseases
Source: PLoS Comput Biol. 2020 Nov 2;16(11):e1008315. doi: 10.1371/journal.pcbi.1008315 (PMC7660930; doi:10.1371/journal.pcbi.1008315)

Tissue

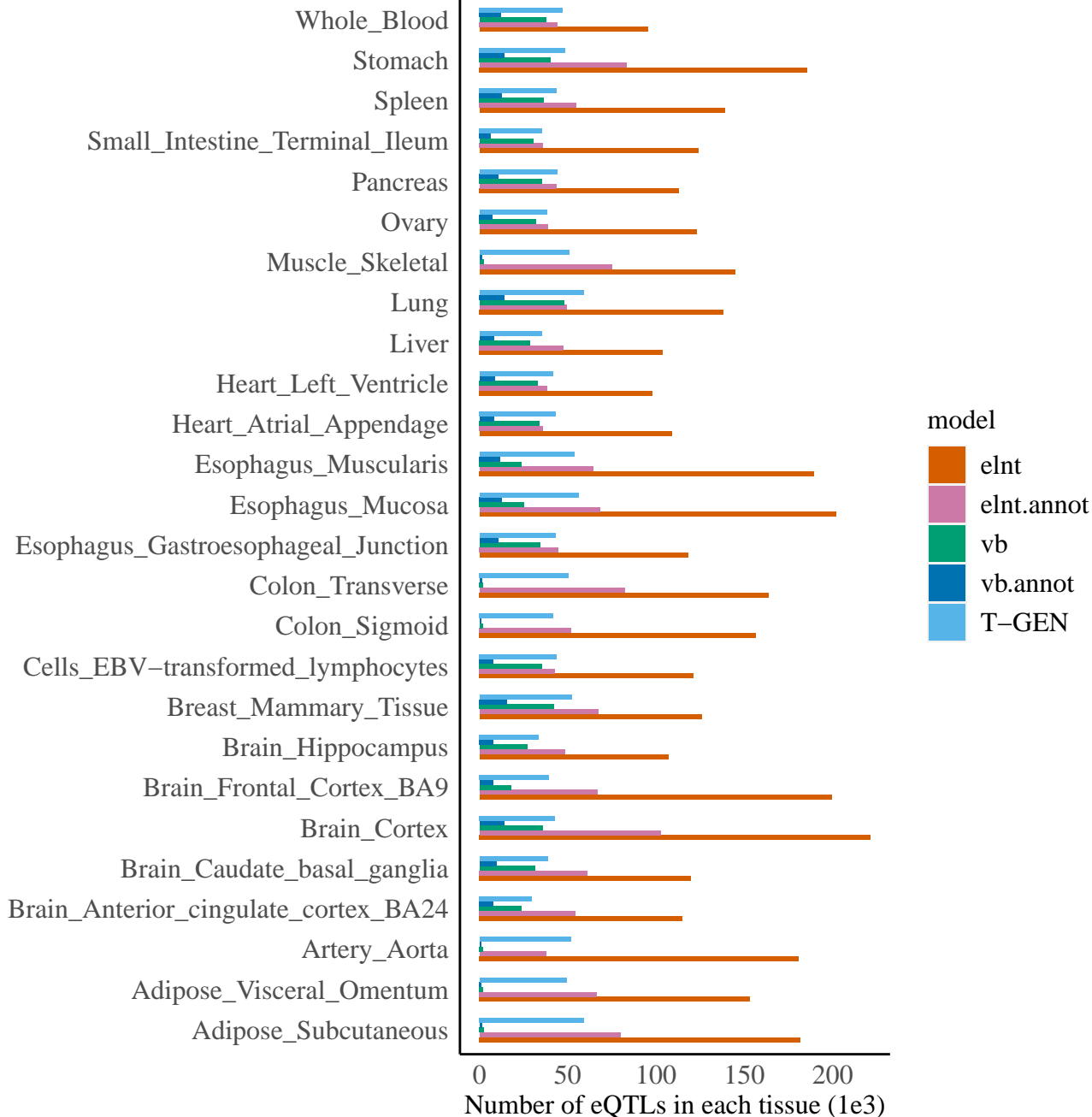

Supplement: S1 Fig — The figure shows the number of identified eQTLs by all five methods across 26 tissues. (PDF) [file pcbi.1008315.s001.pdf]

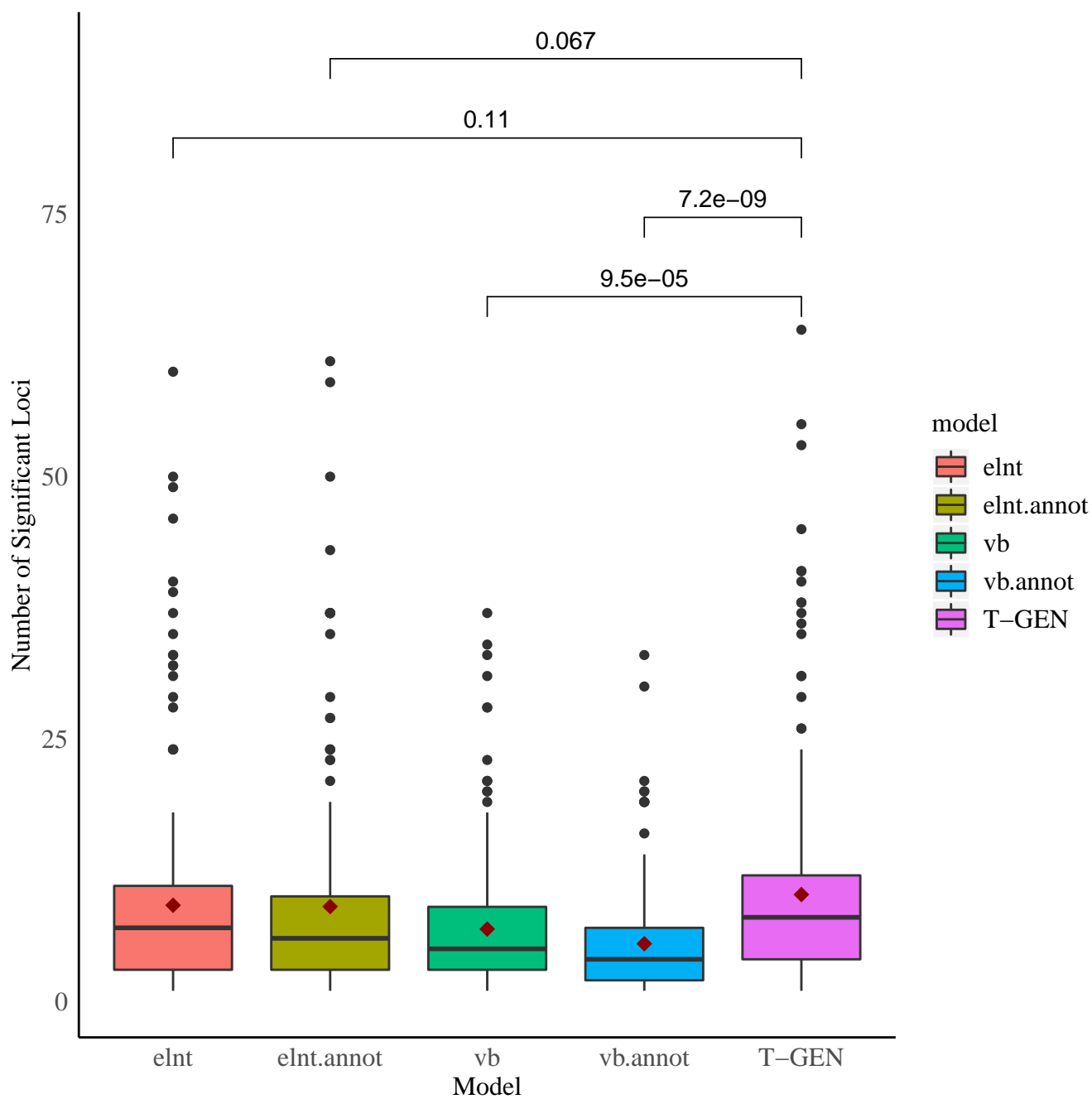

Supplement: S2 Fig — Applied to 207 traits from the LD Hub, significant trait-associated genes were identified in 26 tissues (p-values threshold: 0.05 divided by the number of gene-tissue pairs). Those identified associated-genes were further grouped into pre-defined cytobands. Each boxplot represents the distribution of the number differences between those identified from our tissue-specific analysis and those identified from the four other methods. Y axis is truncated at the value of 3 times the third quarters of each boxplot for visualization. (PDF) [file pcbi.1008315.s002.pdf]

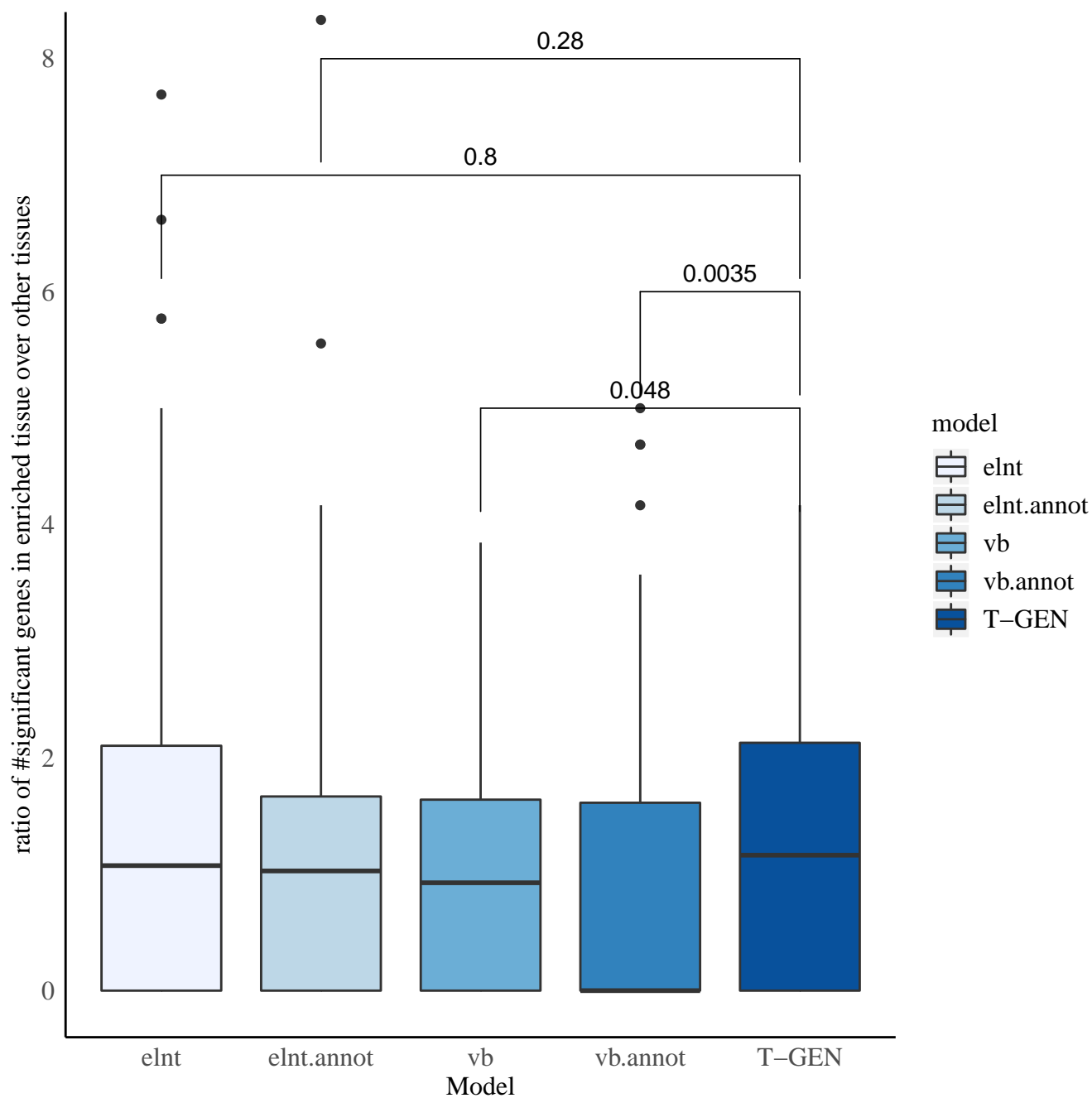

Supplement: S3 Fig — Each boxplot shows the distribution of the ratios across 207 traits in the LD Hub. (PDF) [file pcbi.1008315.s003.pdf]

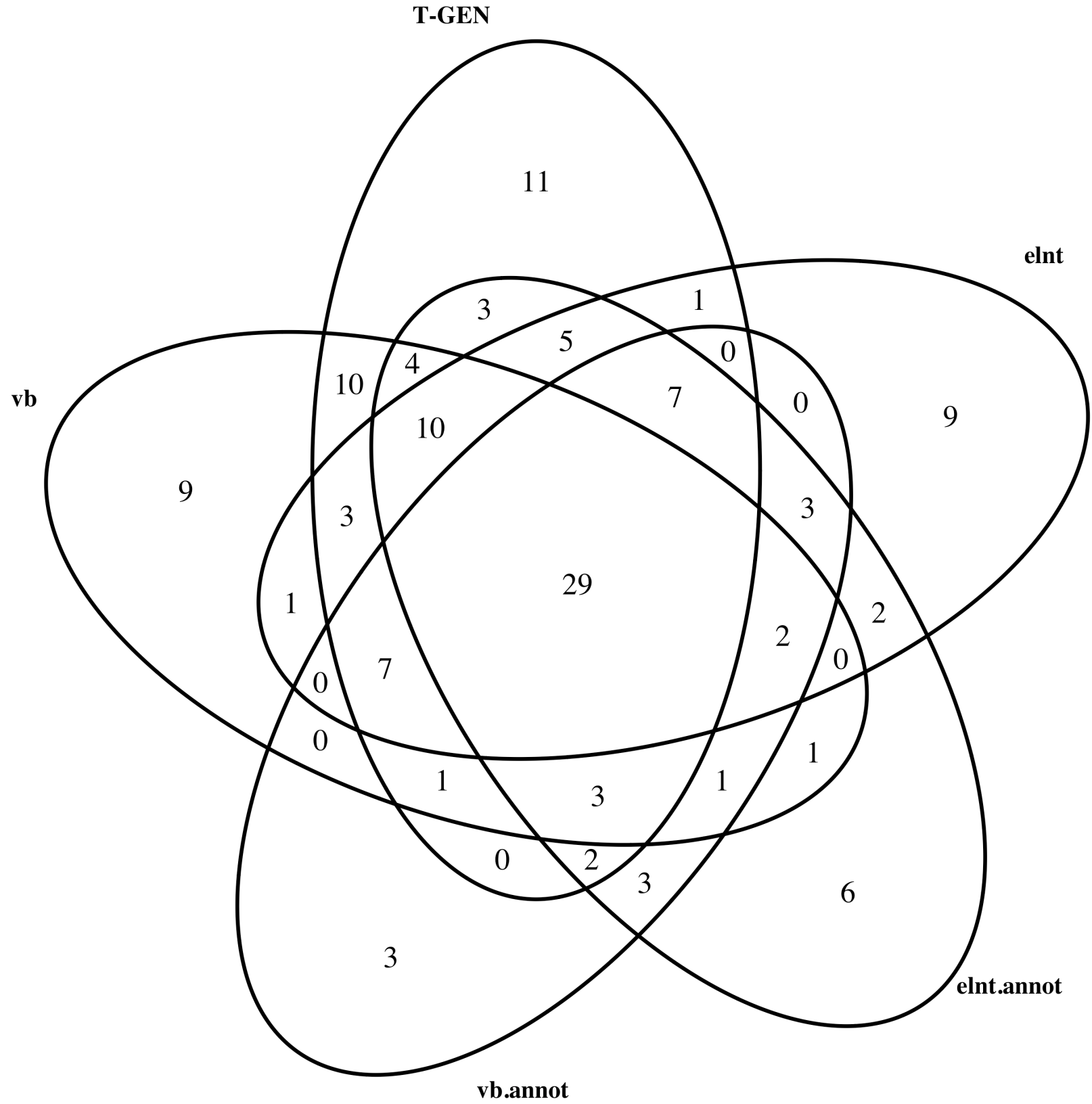

Supplement: S4 Fig — Twenty nine genes were identified by all five methods. 62 genes were shared between T-GEN and elastic-net methods, which may indicate the consistency of gene findings in T-GEN and other TWAS methods based on elastic-net models. (PDF) [file pcbi.1008315.s004.pdf]

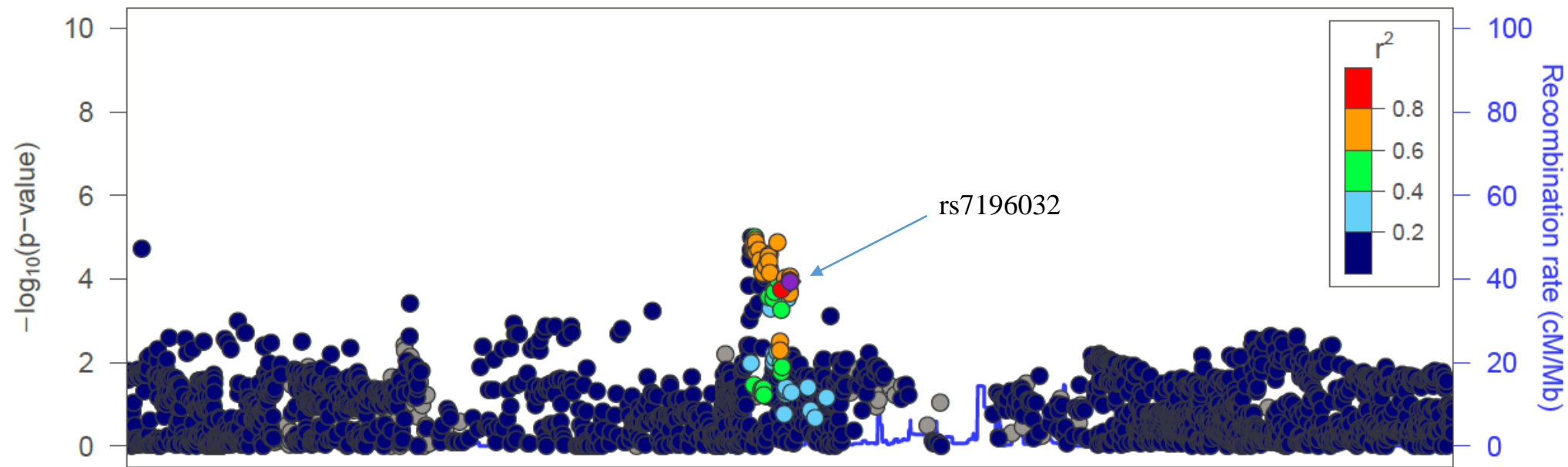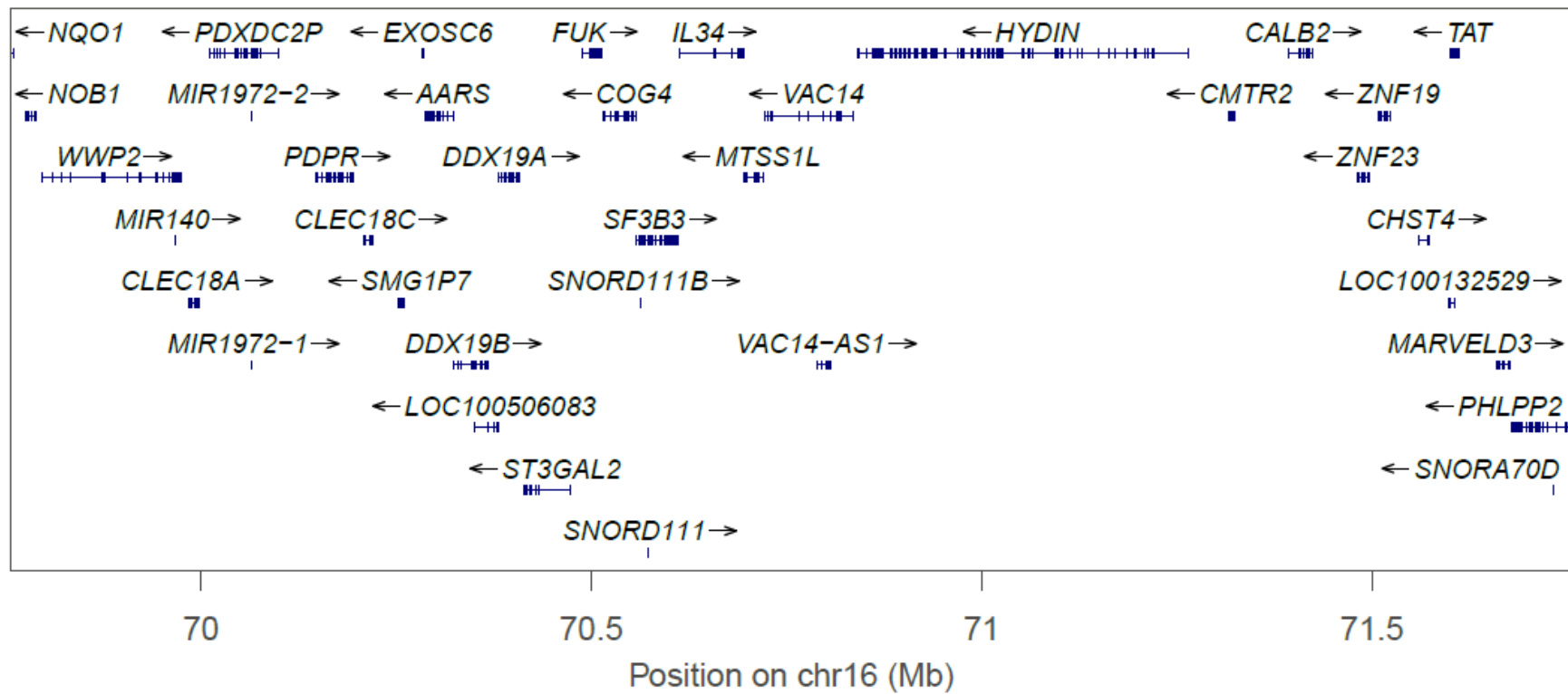

Supplement: S5 Fig — The listed SNP (rs7196032) is one of eQTLs identified by T-GEN in the imputation model of COG4. (PDF) [file pcbi.1008315.s005.pdf]

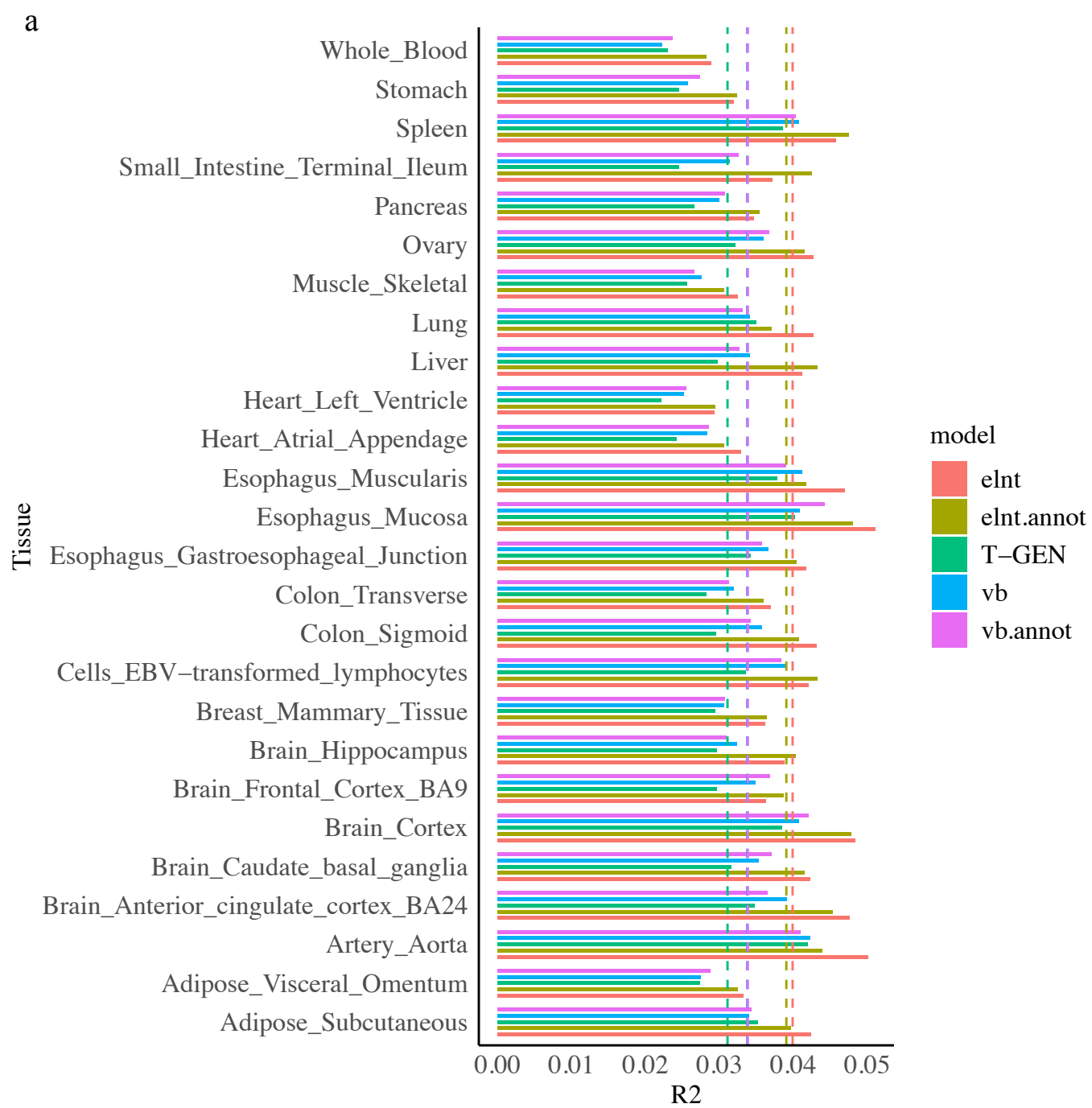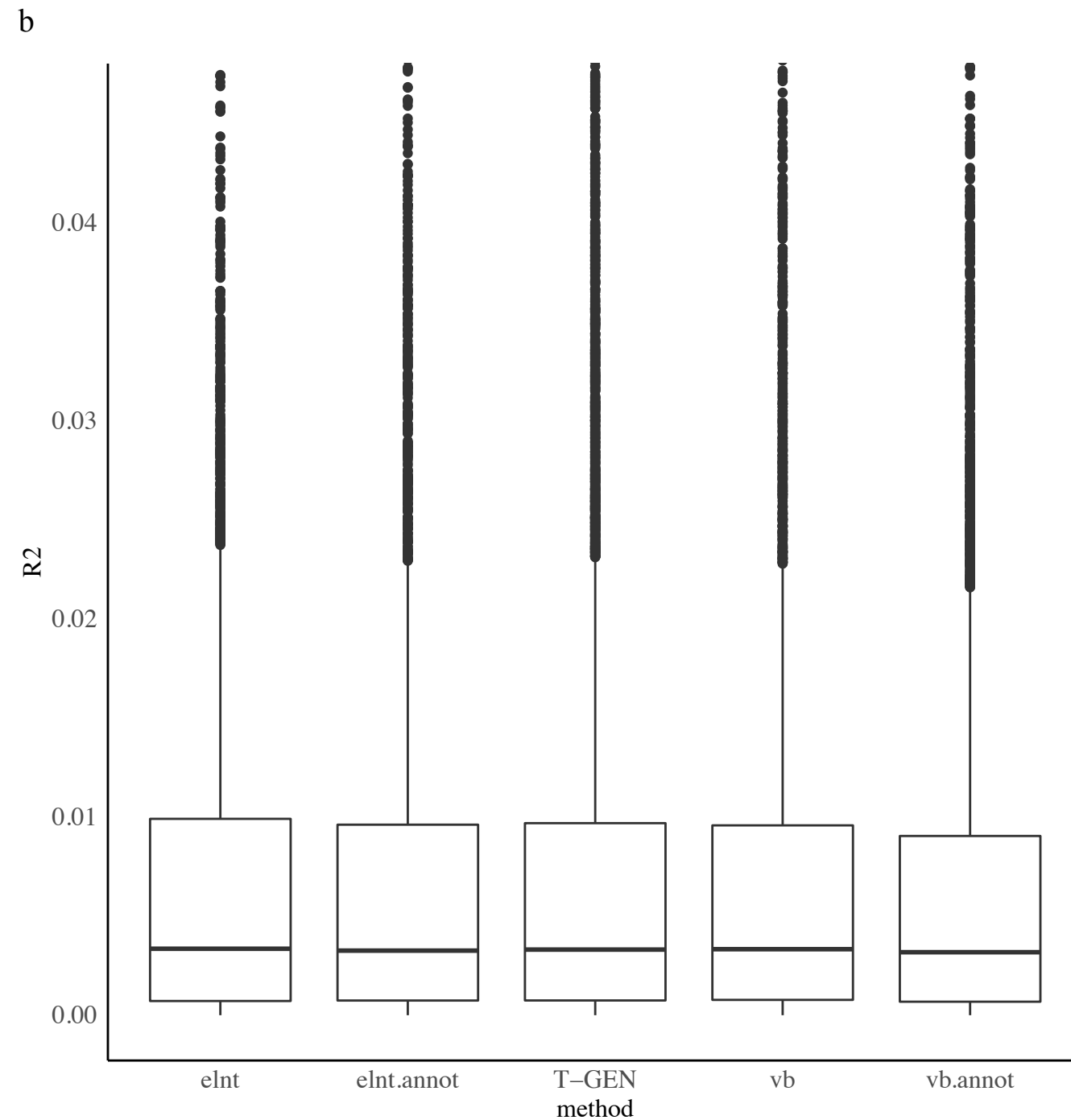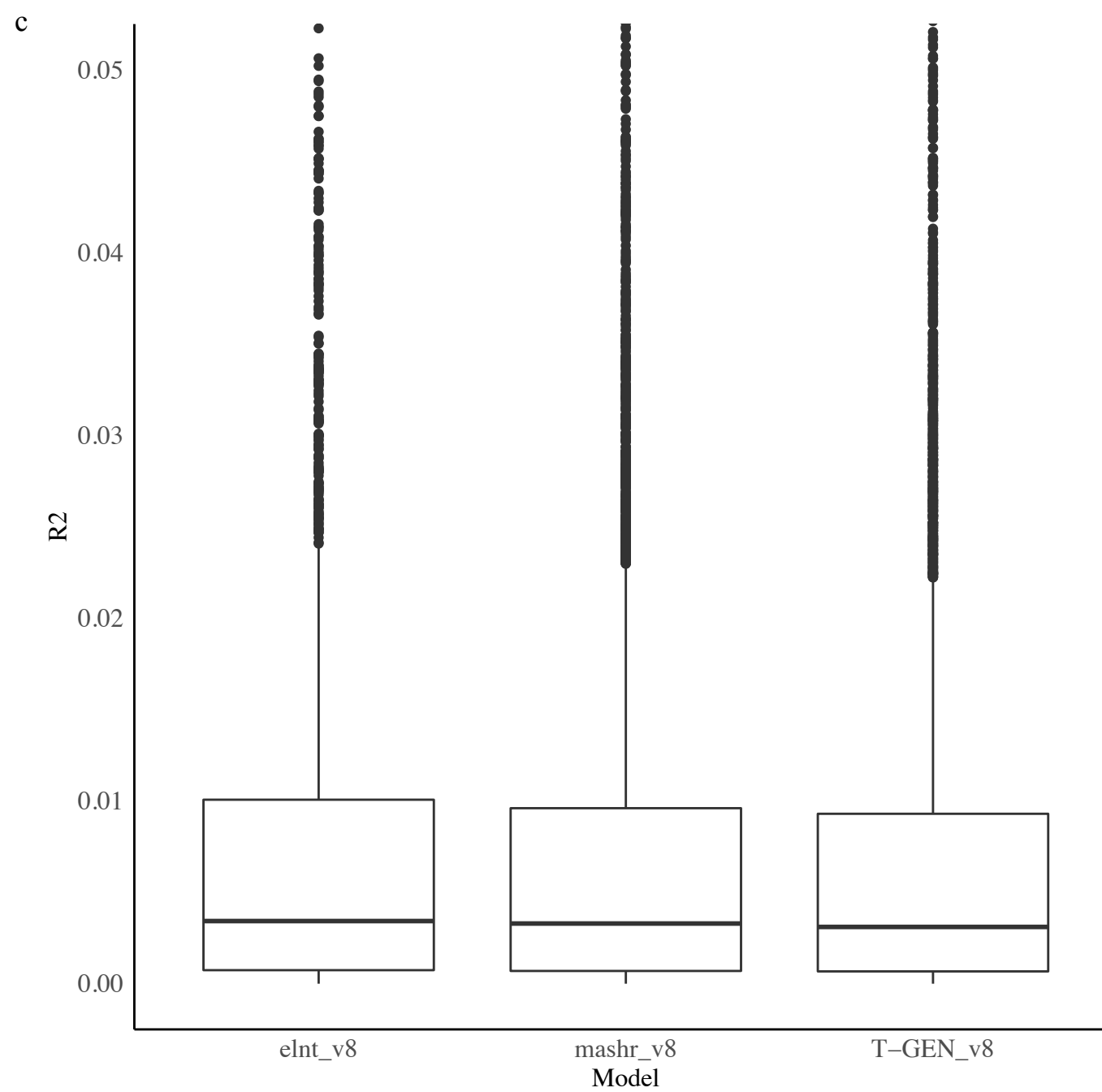

Supplement: S6 Fig — a) indicates the comparison of R2 between observed and imputed gene expression levels in 5-fold cross validation analysis. Using 5-fold cross validation in the GTEx data, R2 between imputed and observed expression levels was calculated in elastic net (elnt) and vb.logit models. Dotted lines indicate the mean values of R2 for each method across all tissues. The mean values of vb.annot and T-GEN models are very close to each other, which lead to the overlaid green and blue dotted lines. b) shows R2 between observed gene expression in the CommonMind dataset and predicted gene expression based on five different methods. T-GEN showed the lowest values of R2 among all five methods. Y axis is truncated at the value of 2 times the third quarters of each boxplot for visualization. c) Using GTEx v8 data, T-GEN, elnt and mashr models were trained in the brain cortex BA9 tissue. R2 between observed gene expression in the CommonMind dataset and predicted gene expression were compared across these three methods. (PDF) [file pcbi.1008315.s006.pdf]

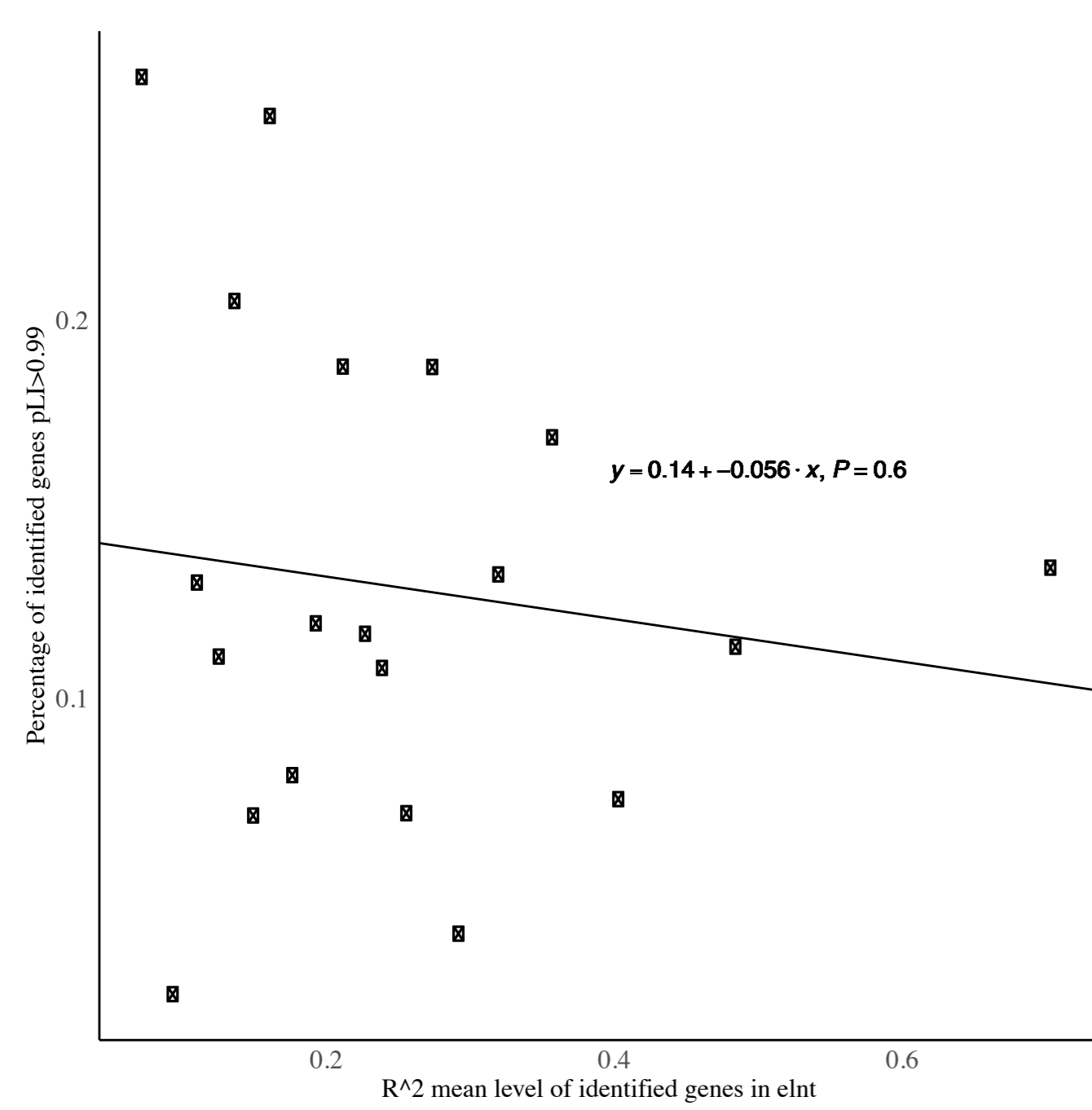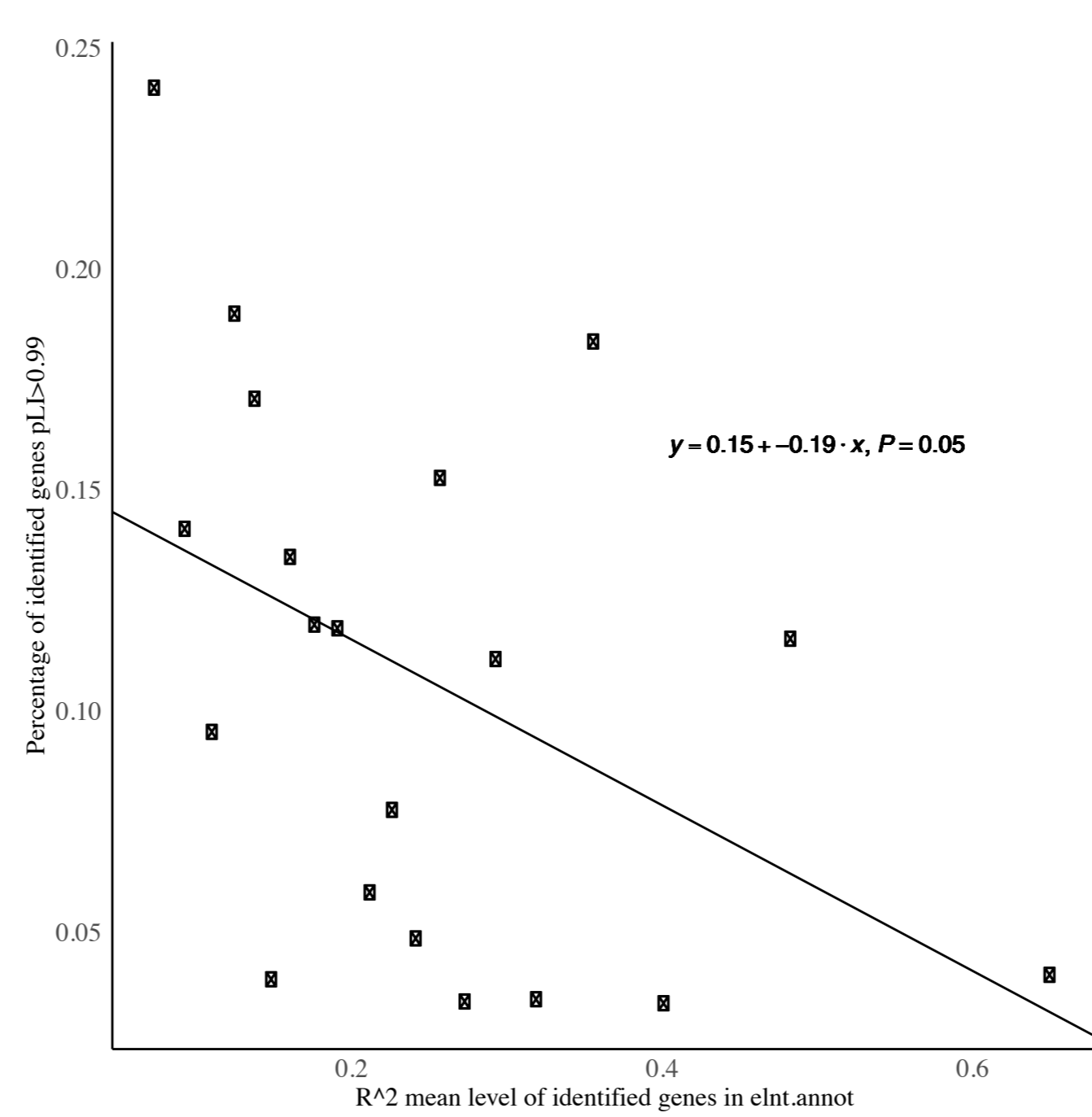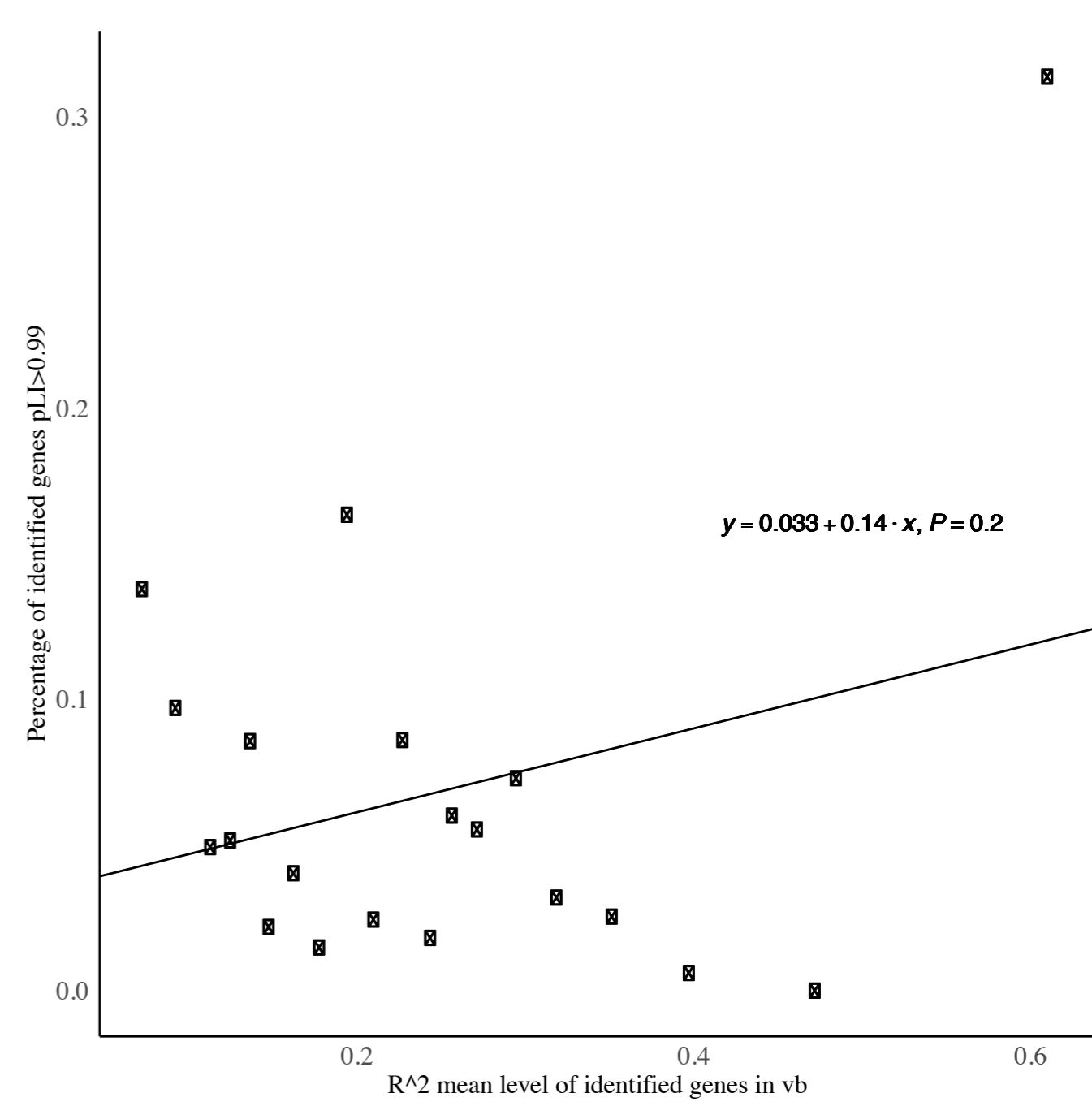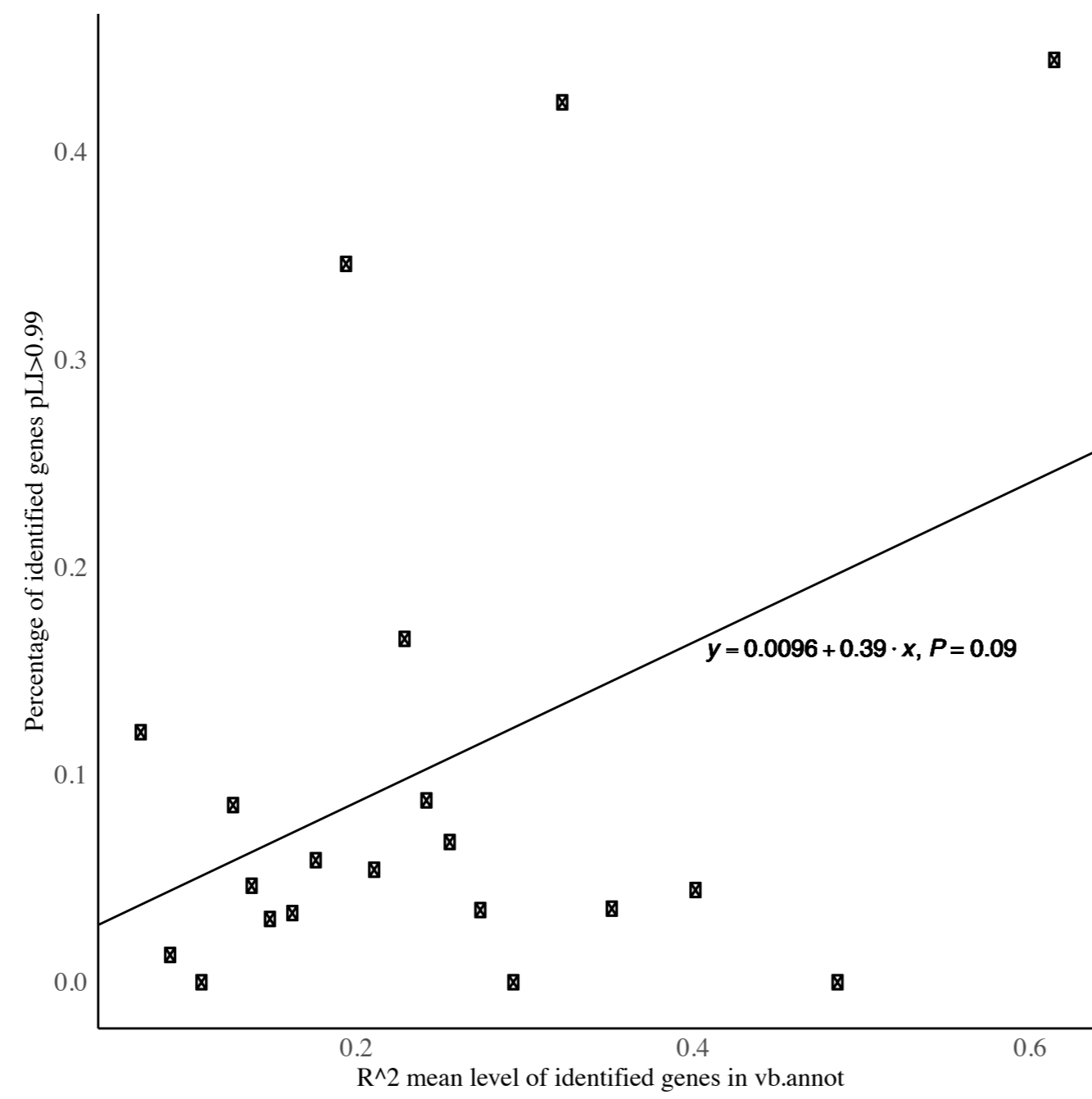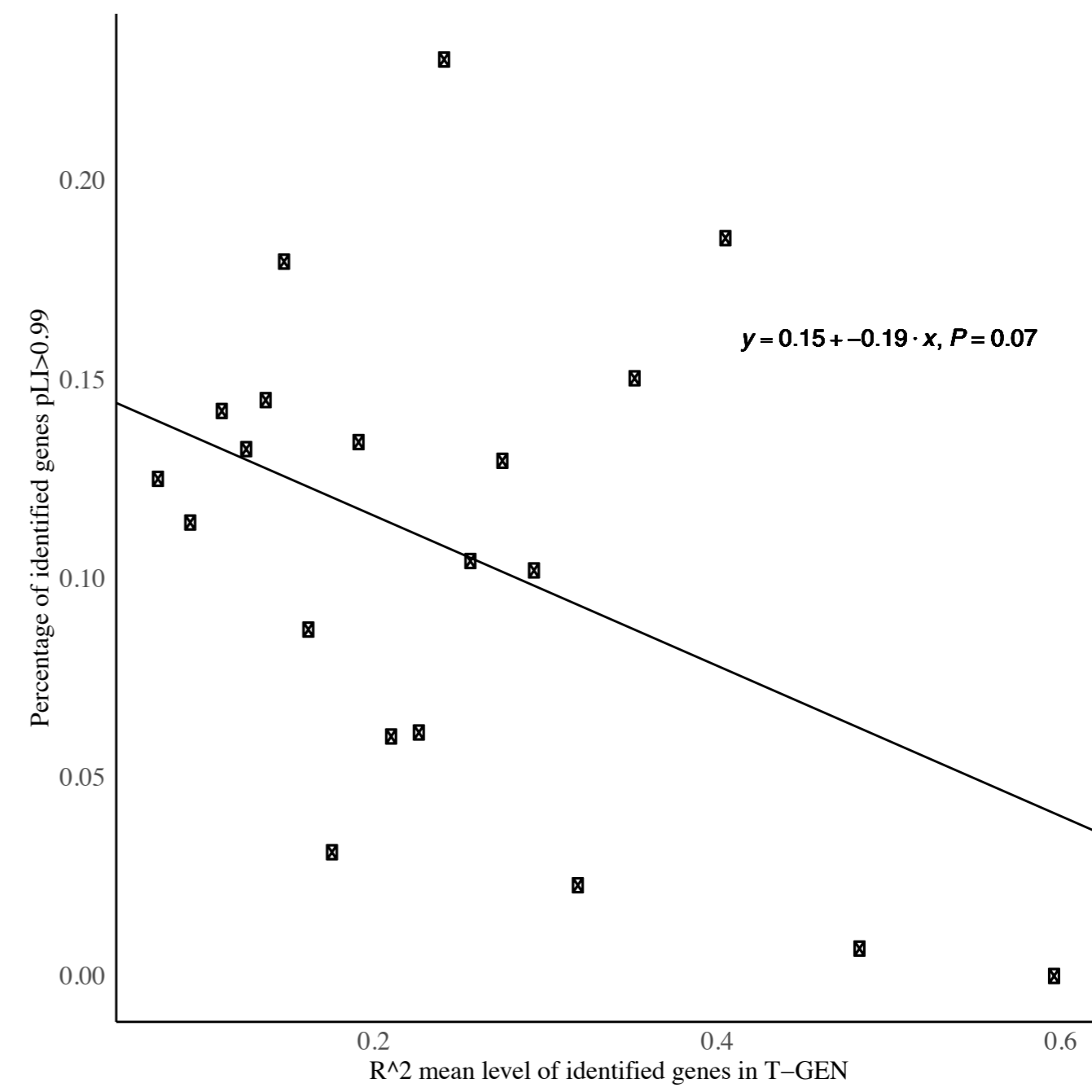

Supplement: S7 Fig — For each method, all trait-associated gene-tissue models are classified into 20 bins based on their imputation accuracy (R2). For each bin, the percentage of genes having pLI>0.99 is calculated. The linear equation indicates the linear relationship between the mean R2 and the percentage and the p value indicate the significance level of the association. (PDF) [file pcbi.1008315.s007.pdf]

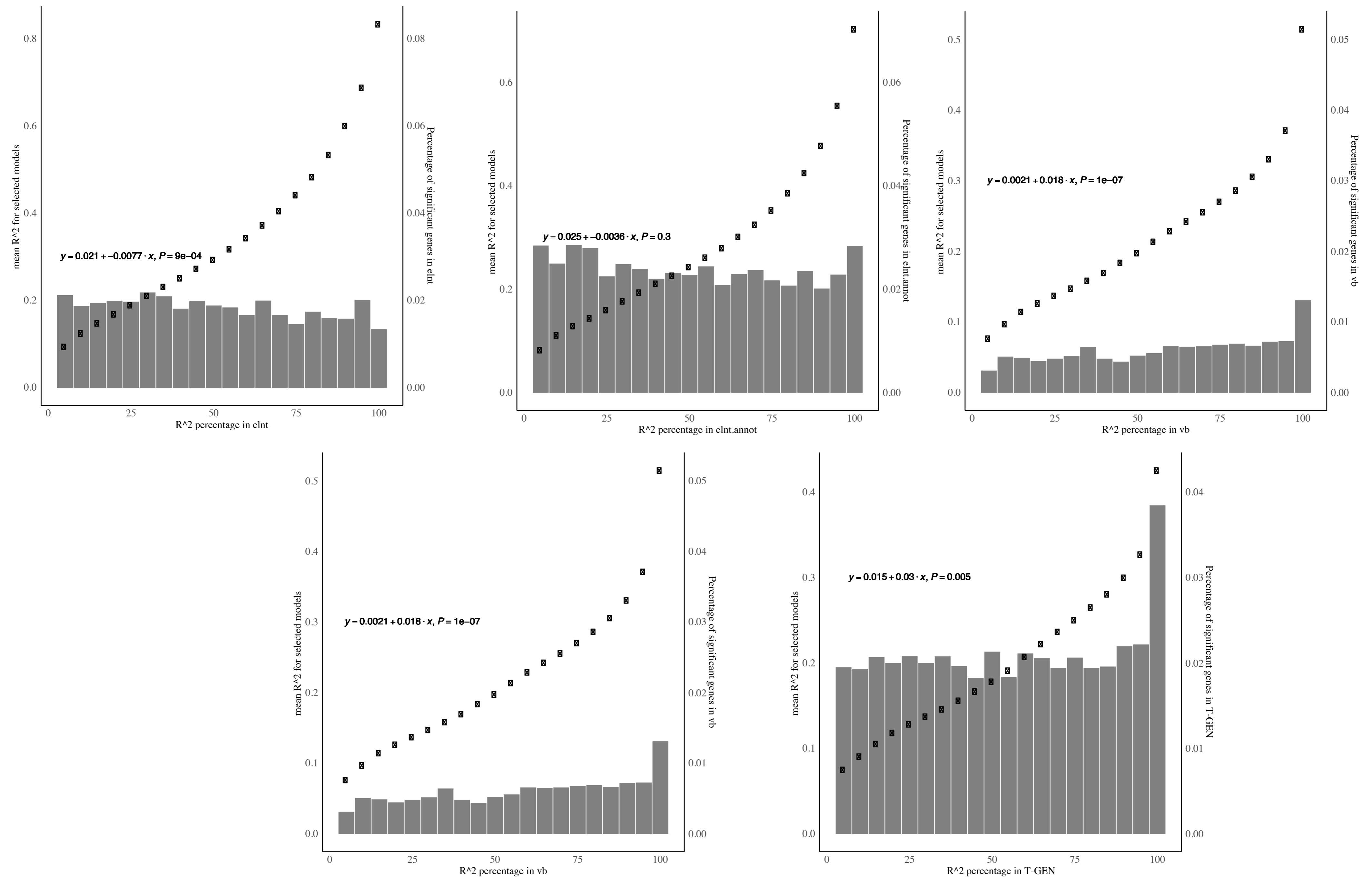

Supplement: S8 Fig — For each method, all gene-tissue models (not just trait-associated ones) are classified into 20 bins based on their imputation accuracy (R2). For each bin, the percentage of gene-tissue models identified as trait-associated (bars) and the mean level of imputation accuracy for trait-associated ones (squares with crosses) are calculated. The linear equation indicates the linear relationship between the mean imputation accuracy and the percentage of trait-associated gene-tissue models. The p value indicates the significance level. (PDF) [file pcbi.1008315.s008.pdf]

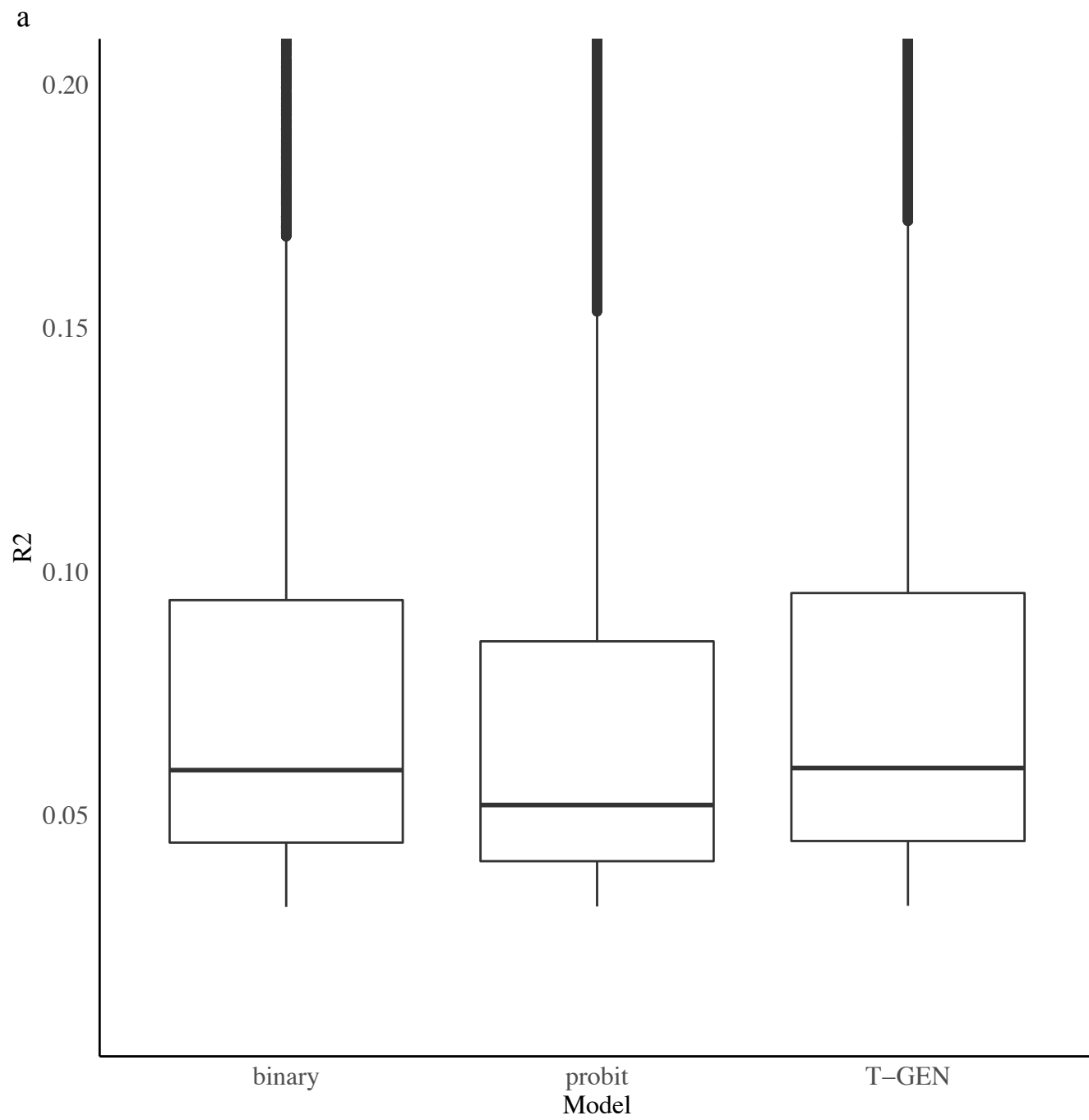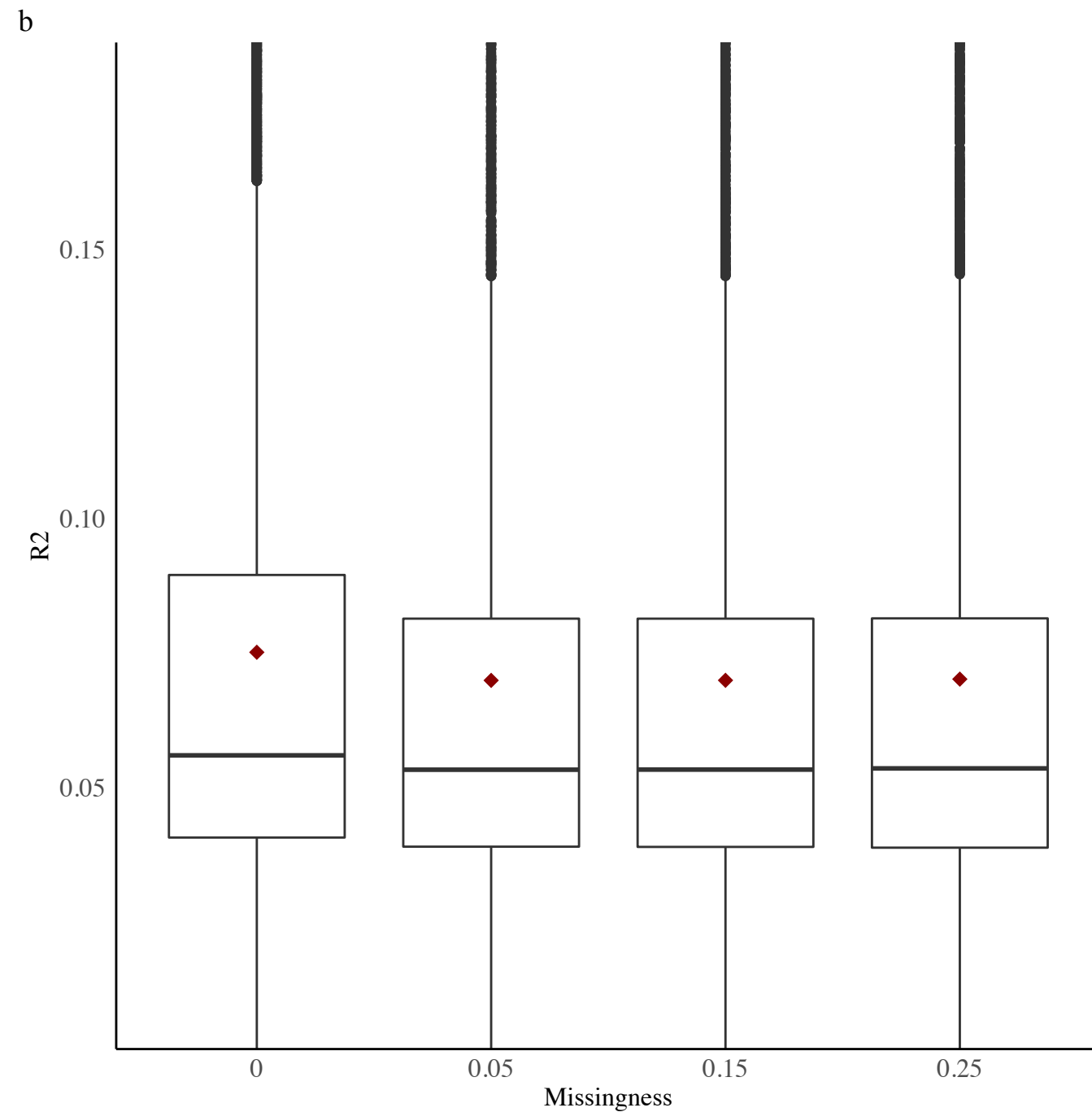

Supplement: S9 Fig — A) shows the comparision among imputation models using binary-coded annotation, models trained using the probit link function in the annotation layer and models trained using the original T-GEN method. b) shows the results of models trained using annotation information with different missing rates. The rate 0 indicates the results of the original T-GEN method. Red diamonds indicate the mean level of each group. (PDF) [file pcbi.1008315.s009.pdf]
