# Supplementary material for: Leveraging functional annotation to identify genes associated with complex diseases
Source: PLoS Comput Biol. 2020 Nov 2;16(11):e1008315. doi: 10.1371/journal.pcbi.1008315 (PMC7660930; doi:10.1371/journal.pcbi.1008315)
Supplement: S1 Text — In the supplementary method part, we showed the details of the variational method used in our T-GEN model including the updating procedures of parameters. In the supplementary discussion part, the influences of gene expression imputation accuracy on gene-trait association test is discussed. Also, the effects of different link function in the annotation layer of T-GEN model, ways of configuring annotation information and incompleteness of annotation were discussed. (DOCX) [file pcbi.1008315.s023.docx]

Supplementary methods

**Variational Bayes model for variable selection in imputing gene expression**

Prior assumptions on hyper parameters

In practice, we do not have any prior information to select the values of $a,b,c,d,a_{0}$ and $b_{0}$, especially for $a_{0}$ and $b_{0}$. Therefore, twenty grid points of $\pi$ are initially sampled from the following distribution, as what have been proposed in the literature [1,2]:

$$\log\left( \frac{\pi_{k}}{1-\pi_{k}} \right)\sim unif(-\log_{10} \left( p \right), -1)$$

*Updating beta coefficients*:

For updating the SNP coefficient $\beta_{k}$ of the SNP $k$ in the model, we would get:

$$\ln Q^{*}\left( \beta_{k} \right)=E_{rest}\left[ \ln P\left( Y | \beta,\epsilon,X \right)+\ln P\left( \beta| \sigma_{\beta}^{2},\sigma^{2},\gamma\right) \right]+constant$$

$$=E_{rest}\left[ \sum_{i=1}^{n} -\frac{\left( y_{i}-x_{i}\beta\right)^{2}}{2\sigma^{2}}+\sum_{j=1}^{p} -\frac{\gamma_{j}\beta_{j}^{2}}{2\sigma_{\beta,j}^{2}\sigma^{2}} \right]+constant$$

$$=E_{rest}\left[ \sum_{i=1}^{n} -\frac{\left( y_{i}-x_{i}\beta\right)^{2}}{2\sigma^{2}}-\frac{\gamma_{k}\beta_{k}^{2}}{2\sigma_{\beta,k}^{2}\sigma^{2}} \right]+constant$$

Therefore, by taking the expectation of the other parameters, $\beta_{k}$ approximately follows a normal distribution where:

$$s_{k}^{2}=Var\left( \beta_{k} | \gamma_{k}=1 \right)= \frac{\sigma^{2}}{\left( X^{T}X \right)_{kk}+1/\sigma_{\beta}^{2}}$$

$$\mu_{k}=E\left( \beta_{k} | \gamma_{k}=1 \right)=\frac{s_{k}^{2}}{\sigma_{k}^{2}}[\left( X^{T}X \right)_{k}-\sum_{j\neq k} \left( X^{T}X \right)_{jk}\alpha_{j}\mu_{j}]$$

$$\alpha_{k}=P\left( \gamma_{k} =1 \right|rest)$$

*Updating the probability of being selected*

The probability of the kth SNP getting selected is dependent not only on the relationship between the SNP genotype and phenotype (gene expression level here), but also on the epigenetic signals of the SNP, which can be described through the equation below:

$$\ln Q^{*}\left( \gamma_{k} \right)=E_{rest}\left[ \ln P\left( Y | X,\beta,\sigma^{2} \right)+\ln P\left( \beta| \gamma,\sigma_{\beta}^{2},\sigma^{2} \right)+\ln P\left( \gamma| A,\omega\right) \right]+constant$$

After solving the equation above term by term, we have:

$$\ln Q^{*}\left( \gamma_{k}=1 \right)=\frac{\mu_{k}^{2}}{2s_{k}^{2}}+\frac{1}{2}\ln\sigma^{2}\sigma_{\beta}^{2}+\ln\frac{1}{1+\exp\left( -A_{k}\omega\right)}+constant$$

$$\ln Q^{*}\left( \gamma_{k}=0 \right)=\ln\frac{1}{1+\exp\left( A_{k}\omega\right)}+constant$$

$$\frac{\alpha_{k}}{1-\alpha_{k}}=\exp\left( \frac{\mu_{k}^{2}}{2s_{k}^{2}} \right)\times\frac{1}{\sigma_{\beta}^{2}\sigma^{2}}\times\frac{1+\exp(A_{k}\omega)}{1+\exp({-A}_{k}\omega)}$$

*Updating coefficients in the logit link of epigenetics signals*

Parameters in the logit link are related to both the SNP epigenetic signals and the status of the SNP being selected or not in last updating step which shown in the following:

$$\ln Q^{*}\left( \omega\right)=E_{rest}\left[ \ln P\left( \gamma| A,\omega\right)+\ln P\left( \omega| \eta\right) \right]+constant$$

Similar to the idea of using variational Bayes method in a logistic regression introduced in the literature [3], $\omega$ also has a normal distribution with parameters:

$$\omega_{N}=E\left( \omega| rest \right)=V_{N}\sum_{j=1}^{p} \frac{\gamma_{j}}{2}A_{j}^{T}$$

$$V_{N}^{-1}=\frac{1}{Var(\omega|rest)}=E_{rest}\left( \eta\right)I+2\sum_{j=1}^{p} \lambda\left( \xi_{j} \right)A_{j}^{T}A_{j}$$

To calculate the overall PPS, we need to get the likelihood of the full model under each prior parameter setting. Since we actually approximate the largest value of the lower bound in the full model likelihood, the estimated optimal lower bound is used to substitute for the exact likelihood.

$$\ln P\left( Y | X,Q,A \right)\geq F\left( Q,\theta\right)$$

$$=\int\int\int q(\beta,\gamma,\omega;\theta)log \frac{P(Y,\beta,\gamma,\omega|X,A;\theta)}{q(\beta,\gamma, \omega;\theta)}d\beta d\gamma d\omega$$

$=E_{\beta,\gamma, \omega}\left[ \log P\left( Y,\beta,\gamma,\omega| X,A;\theta\right) \right]-E_{\beta,\gamma, \omega}[\log q(\beta,\gamma,\omega|\theta)]$

where

$$\log P\left( Y,\beta, \gamma,\omega| X,A,\theta\right)=\log P\left( Y | \beta,\sigma^{2} \right)+\log P(\beta|\gamma,\sigma^{2},\sigma_{\beta}^{2})+\log P\left( \gamma| \omega,A \right)+\log P(\omega|\eta)$$

$$\log q\left( \beta,\gamma,\omega;\theta\right)=\log q\left( \beta| \gamma;\theta\right)+\log q\left( \gamma| \omega;\theta\right)+\log q(\omega;\theta)$$

Then, the lower bound becomes:

$$F\left( Q,\theta\right)=-\frac{n}{2}\log2\pi\sigma^{2}-\frac{\left| \left| Y-X\alpha\mu\right| \right|^{2}}{2\sigma^{2}}-\frac{1}{2\sigma^{2}}\sum_{j=1}^{P} [\left( X^{T}X \right)_{jj}Var(\beta_{j})]$$

$$-\sum_{j=1}^{P} \alpha_{j}\log\frac{\alpha_{j}}{\pi}-\sum_{j=1}^{P} \left( 1-\alpha_{j} \right)\log\frac{1-\alpha_{j}}{1-\pi}$$

$$+\sum_{j=1}^{p} \frac{\alpha_{j}}{2}(1+\log\frac{s_{k}^{2}}{\sigma^{2}\sigma_{\beta}^{2}}-\frac{s_{k}^{2}+\mu_{k}^{2}}{\sigma^{2}\sigma_{\beta}^{2}})$$

$$+\frac{1}{2}w_{N}^{T}v_{N}^{-1}w_{N}+\frac{1}{2}\ln\left| V_{N} \right|+\sum_{j=1}^{P} [-\ln\sigma\left( \xi_{j} \right)-\frac{\xi_{j}}{2}+\lambda\left( \xi_{j} \right)\xi_{j}^{2}]$$

$$-\ln\Gamma\left( a_{0} \right)+a_{0}\ln b_{0}-b_{0}\frac{a_{N}}{b_{N}}+\ln\Gamma\left( a_{N} \right)+a_{N}$$

The implementation of the method utilized part of the script used in the varbvs R package [1]. The running time of our method is longer than that of elastic net (glmnet R package) [4] when fitting models on a data set with 100 individuals, 1,000 SNPs and 5 kinds of annotation array. Our method takes 15.15 minutes while fitting models repeated for 100 times while the elastic net takes 13.90 seconds on the same CPU (Intel(R) Xeon(R) CPU E5-2620_v3 2.40GHz). More specifically, the running time for modeling training in each tissue was displayed in the **S11 Table**. The computation time provided in the supplement text serves a better representation since the computation jobs of each tissue may be assigned to computation nodes with different properties, of which we could not keep full track.

Supplementary Discussion

**Gene expression imputation accuracy and gene-trait association test**

Gene expression imputation accuracy was compared across all five methods through both five-fold cross validation and prediction in an external dataset from the CommonMind Consortium ([www.synapse.org/CMC](http://www.synapse.org/CMC)). We compared the $R^{2}$ (squared correlation) between the observed and imputed expression levels of our method with that from the other methods. In five-fold cross-validation analysis using the GTEx data (**S6a Fig**), our method showed $R^{2}$ decrease from 6.3% (-2.4e-3,compared with vb.annot) to 22.06% (7.2e-3,compared with elastic net), compared to the other four methods (Fig 5, supplementary). For prediction analysis in the CommonMind dataset, expression imputation models trained on the GTEx Brain Cortex BA9 tissue were used to predict gene expression levels based on individual genotype data of CommonMind. All five methods had similar $R^{2}$ (~0.007) between predicted gene expression and whole expression in the Brain Cortex BA9 tissue of CommonMind (**S6b Fig**) but we note that our method included more genes whose expression levels can be predicted. For all five methods, in-sample cross-validation performance was much better than that in out-sample prediction as expected. To further evaluate how the sample size of training data affects the prediction accuracy in the external CommonMind data, we also compared the performance of imputation models trained using the latest release of GTEx data (v8). We have compared prediction accuracy of models trained by T-GEN, prediXcan (elastic net) and mash. All three models were built using GTEx v8 model. The prediction accuracy did not show significant improvements with PrediXcan (v8) models having $R^{2}$ of 0.0076 (elnt v6: 0.0072, p=0.09), mash (v8) models having $R^{2}$ of 0.0073 and T-GEN models having $R^{2}$ of 0.0070 (T-GEN v6: 0.0071, p=0.11).(**S6c Fig**).

We further investigated the impact of imputation accuracy on the identification of disease-associated genes. We did not observe a consistent pattern between imputation accuracy and the percentage of associated genes identified with pLI>0.99 across the five methods (**S7 Fig**). Similarly, the percentage of genes identified associated with 207 traits did not show a consistent pattern with imputation accuracy across five methods either (**S8 Fig**).

**Gene expression imputation accuracy and annotation layer**

To evaluate the impacts of different annotation formulations, we constructed binary function annotations by assigning any positive annotation values as 1 and others as 0. To alleviate the potential impacts of limited sample sizes here, we used GTEx v8 data in the muscle skeletal tissue that has the largest samples size (n=706) across all the tissues for the following analysis. The squared Pearson correlation ($R^{2}$) between imputed expression and measured expression was calculated when using either binary or continuous annotations (**S9a Fig**). Models trained using continuous annotations ($R^{2}:0.075$) showed slightly better (p=0.03) imputation accuracy than those using binary annotations ($R^{2}:0.072$) and identified a slightly higher percentage (continuous: 0.68%; binary: 0.65%) of potentially functional SNPs (CADD score > 20).

We also compared models using the probit link function in the annotation layer with models trained using the logistic link in the annotation layer. The use of different link functions mainly affects the update process of probabilities of SNPs getting selected and that of coefficients in the annotation layer. We mainly followed the work by Albert and Chib [5] to compare these two link functions (**S9a Fig**) and found that a significant (p=5.56e-3) but modest level of improvement of the logit-link ($R^{2}:0.075$) over the probit-link ($R^{2}:0.073$) function in imputation accuracy. Also, the logit-link model identified a slightly higher fraction (0.68% vs. 0.63%) of functional SNPs (CADD score>20). One potential reason behind the better performance of logit-link used here is that the logit link function may be less affected by extreme predictors compared to the probit link function [6].

By considering different proportions (5%, 15%, 25%) of SNPs with missing annotations, where we set missing the annotation values as 0, we further evaluated the impact of incompleteness of annotations. Different levels of incompleteness did not show significant impact on imputation accuracy (**S9b Figure**) though models built with the full annotation information achieved the best accuracy ($R^{2}$) of 0.075 (5% missing: 0.07, 15% missing: 0.07, 25% missing: 0.0702). As the missing rate increased, the fraction of SNPs with CADD scores larger than 20 decreased (original: 0.69%; 5% missing: 0.64%; 15% missing: 0.57%; 25% missing: 0.53%). Only when compared with models with the highest proportion of missing annotation 25%, T-GEN with full annotation information showed a statistically significant difference (p=4e-3) in identifying SNPs with CADD scores > 20. Overall, annotation missingness did affect T-GEN performance with moderate level of missingness.

**References:**

1. Carbonetto P, Stephens M. Scalable Variational Inference for Bayesian Variable Selection in Regression, and Its Accuracy in Genetic Association Studies. Bayesian Anal. 2012;7: 73–108. doi:10.1214/12-BA703

2. Zhou X, Carbonetto P, Stephens M. Polygenic Modeling with Bayesian Sparse Linear Mixed Models. PLOS Genet. 2013;9: e1003264. Available: https://doi.org/10.1371/journal.pgen.1003264

3. Drugowitsch J. Variational Bayesian inference for linear and logistic regression. arXiv Prepr arXiv13105438. 2013.

4. Friedman J, Hastie T, Tibshirani R. Regularization Paths for Generalized Linear Models via Coordinate Descent. J Stat Softw. 2010;33: 1–22. Available: https://www.ncbi.nlm.nih.gov/pubmed/20808728

5. Albert JH, Chib S. Bayesian Analysis of Binary and Polychotomous Response Data. J Am Stat Assoc. 1993;88: 669–679. doi:10.1080/01621459.1993.10476321

6. Zou H, Hastie T. Regularization and Variable Selection via the Elastic Net. J R Stat Soc Ser B (Statistical Methodol. 2005;67: 301–320. Available: http://www.jstor.org/stable/3647580
