# Supplementary material for: Leveraging functional annotation to identify genes associated with complex diseases
Source: PLoS Comput Biol. 2020 Nov 2;16(11):e1008315. doi: 10.1371/journal.pcbi.1008315 (PMC7660930; doi:10.1371/journal.pcbi.1008315)
Supplement: Attachment — Submitted filename: T-GEN response_3.docx [file pcbi.1008315.s024.docx]

We thank all the reviewers for their very valuable and insightful comments. In the revised paper, we have made the following major changes and updated the corresponding results and discussions in the main text and supplementary materials to address the comments.

1. We have updated the T-GEN models using GTEx v8 data in whole blood, liver and brain frontal cortex BA9.

- We have compared the out-sample predictions of T-GEN (v8) using CommonMind data with elnt (v8) and mash (v8) models. See R.2.1, R.2.3, and R.3.3. where R.x.y denotes our response to the yth comment from the xth reviewer.
- We have updated the associated-gene identification results of late-onset Alzheimer’s disease in related tissues, see R.2.3, R.2.7, and R.3.3.

2. We have investigated the impact of using different link functions in gene expression imputation, see R.1.4.
3. We have investigated the impact of using different formulations of annotation in gene expression imputation, see R.1.4.
4. We have investigated the effects of annotation incompleteness in gene expression imputation, see R.3.2.

Reviewer #1: Liu et al introduced a new method called T-GEN (Transcriptome-mediated identification of disease-associated Gens with Epigenetic aNnotation) to identify disease-associated genes leveraging epigenetic information. They incorporate epigenomic annotation in their formula as a Bayes prior (variational Bayes method) in imputing gene expression levels and then perform TWAS (transcriptome-wide association study) of disease traits.

This method leverages functional annotation to identify genes associated with complex diseases. They applied T-GEN to 207 complex traits and identified more trait-associated genes (ranging from 7.7 % to 102%) than those from existing methods. Among the identified genes associated with these traits, T-GEN can better identify genes with high (>0.99) pLI scores (higher scores indicate more functional importance of genes) compared to other methods. When T-GEN was applied to late-onset Alzheimer’s disease, they identified 96 genes located at 15 loci, including two novel loci not implicated in previous GWAS. I believe that the method proposed in this paper is an important extension of existing methods in identifying disease associated genes. The paper has been well written. I have the following comments.

We thank the reviewer for thoughtful review and positive summary of our manuscript. We have carefully gone through the comments and addressed them as follows:

Q.1.1 The authors assumed that SNPs with active epigenetic annotations are more likely to regulate tissue-specific gene expression. I think this is only partially true given our knowledge about human genome. More GWAS SNPs are located in the non-coding and unknown regions. In addition our knowledge about epigenetic annotations is limited, mainly based on prediction by statistical models. Therefore, this assumption is clearly a limitation to the method but may not have a good solution at this point. I wonder if the authors can comment on this and provide some insights in the discussion.

R.1.1. We agree with this reviewer on the limitation in that our proposed model assumes the utility of epigenetic annotations and its performance may be affected by our limited understandings about epigenetic annotations. We have added the following statements about this limitation in the discussion part of the manuscript as follows:

“*Another limitation of our method is that we assume SNPs with known active epigenetic signals are more likely to regulate gene expression while the regulatory effects of epigenetics can be rather complex. For example, H3K9ac can be present in both actively transcribed and bivalent regions* (*BMC Genomics 13, no. 1 (2012): 424). It has also been shown that Cytosine-phosphate diester-guanine (CpG) islands associated with gene expression may have intermediate instead of low methylation levels (BMC Genomics 19, no. 1 (2018): 1-13). Although trait heritability and GWAS risk SNPs are both enriched in genomic regions with active epigenetic signals in related cell types or tissues, most of these studies have been based on in-silico analysis (Science 337, no. 6099 (2012): 1190-1195; Nature Genetics 45, no. 2 (2013): 124-130; The American Journal of Human Genetics 95, no. 5 (2014): 535-552; Nature Genetics 47, no. 11 (2015): 1236; Nature Genetics 50, no. 10 (2018): 1483-1493). Using more data on epigenetic regulation collected from biological experiments instead of in-silico predictions can further improve the power of our approach*.”

Also, we have added notes on published experimental studies that lend support for the importance of epigenetic annotations in the introduction part, see our response R.3.1 in the following where the third reviewer raised similar comments:

“*To more accurately identify regulatory eQTLs that play functional roles, we assume that SNPs with active epigenetic annotations are more likely to regulate tissue-specific gene expression [14–18].*”

14.	Geyer PK, Green MM, Corces VG. Tissue-specific transcriptional enhancers may act in trans on the gene located in the homologous chromosome: the molecular basis of transvection in Drosophila. EMBO J. 1990;9: 2247–2256. Available: https://pubmed.ncbi.nlm.nih.gov/2162766

15.	Ong C-T, Corces VG. Enhancer function: new insights into the regulation of tissue-specific gene expression. Nat Rev Genet. 2011;12: 283–293. doi:10.1038/nrg2957

16.	Bell JT, Pai AA, Pickrell JK, Gaffney DJ, Pique-Regi R, Degner JF, et al. DNA methylation patterns associate with genetic and gene expression variation in HapMap cell lines. Genome Biol. 2011;12: R10. doi:10.1186/gb-2011-12-1-r10

17.	Swift-Scanlan T, Smith CT, Bardowell SA, Boettiger CA. Comprehensive interrogation of CpG island methylation in the gene encoding COMT, a key estrogen and catecholamine regulator. BMC Med Genomics. 2014;7: 5. doi:10.1186/1755-8794-7-5

18.	Kumar D, Puan KJ, Andiappan AK, Lee B, Westerlaken GHA, Haase D, et al. A functional SNP associated with atopic dermatitis controls cell type-specific methylation of the VSTM1 gene locus. Genome Med. 2017;9: 18. doi:10.1186/s13073-017-0404-6

Q.1.2. ‘Reported GWAS hits are enriched in regions with active epigenetic signals and they can help fine-map true GWAS hits with functional impacts’. I wonder what proportion of GWAS hits have active epigenetic signals.

R.1.2. Thank you for your question. We have added the following statement in the manuscript “*the Encyclopedia of DNA Elements (ENCODE) project found that 34% of disease-associated SNPs overlap DNA-hypersensitive sites (encompassing 3.9% of the whole genome sequence)*” on page 4 line 95-97.

Q.1.3. What does ‘positive value’ mean in ‘Removing cis-SNPs without positive values for H3K4me1, H3K4me3, or H3K9ac signals’? What are the effects of the SNPs that show positive values with these three genes?

R.1.3. Sorry for the confusion. A positive value for an SNP means that the corresponding genomic region for this SNP has the specific corresponding epigenetic modification, where the higher the value is, the stronger the epigenetic modification is.

Positive values of H3K4me1 and H3K4me3 epigenetic markers indicate active transcriptions of the corresponding genomic regions, where H3K4me1 is often associated with gene enhancers (*Molecular cell* 53, no. 6 (2014): 979-992), and H3K4me3 often occurs in the promoters of actively transcribed genes and is associated with activated gene transcription (*Cell* 152, no. 5 (2013): 1021-1036). H3K9ac is part of the active promoter state, but also exists in bivalent promoters and can possibly promote transcription elongation (*Journal of Biological Chemistry* 292 no.35 (2017): 14456-14472).

Q.1.4. How did the author form a continuous epigenetic annotation score? Did they compare different ways to formulate the annotation score? How did the author choose the current method to form continuous annotation? Is there an advantage to use logit link to associate the epigenetic annotation with the probability of a SNP being an eQTL?

R.1.4. We took the output from the Roadmap Epigenomics Project as the continuous annotation score, which is the ratio of ChIP-seq or DNase counts relative to the expected background counts for these two types of annotations, and the fraction of methylation reads for DNA methylation signal. We have clarified this in the method part of the manuscript as

“*We only consider epigenetic signals for SNPs which have significant epigenetic markers (p<1e-2). For SNPs that don’t have significant epigenetic markers, we set their corresponding annotation to be 0*.” Compared to using binary annotations, continuous annotations may be more informative for SNP selection.

We have compared the performance of using continuous annotations with that using binary annotations which treats significant positive values as 1 and others as 0. We have also compared the performance of the model using the probit link as well. The results are provided in the S1 Text as follows:

“*To evaluate the impacts of different ways of formulating annotation information, we constructed binary functional annotations by assigning any positive annotation value as 1 and others as 0. To alleviate the potential impacts of limited sample sizes in developing gene expression imputation models, for the following analyses, we considered the muscle skeletal tissue in the GTEx v8 data as this tissue has the largest sample size (n=706) across all the tissues. We calculated the squared Pearson correlation* *(*$R^{2}$*) between imputed expression and measured expression using either binary or continuous annotations (****S9a Fig****). Models trained using continuous annotations (*$R^{2}:0.075$*) showed slightly better (p=0.03) imputation accuracy than those using binary annotations (*$R^{2}:0.072$*), and identified a slightly higher percentage (continuous: 0.68%; binary: 0.65%) of potentially functional SNPs (CADD score > 20).*

*We also compared models using the probit link function in the annotation layer with models trained using the logistic link function. The use of different link functions mainly affects the updates of the probabilities of SNPs being selected and the coefficients in the annotation layer. We mainly followed the work by Albert and Chib (Journal of the American statistical Association 88, no. 422 (1993): 669-679) to compare these two link functions (****S9a Fig****), and found a modest but significant (p=5.56e-3) improvement of the logit-link (*$R^{2}:0.075$*) over the probit-link (*$R^{2}:0.073$*) in imputation accuracy. Also, the logit-link model identified a slightly higher fraction (0.68% vs 0.63%) of functional SNPs (CADD score>20). One potential reason for the better performance of the logit-link function may be that the logit link can be less affected by extreme predictors compared to the probit link function.”*

We note that it has been observed in the literature that the logit link function can be less affected by extreme predictors than the other link functions such as the probit link function (*The Journal of the Royal Statistical Society, Series B* (2005): 1-12). Here we didn’t normalize the epigenetic annotations since the scale of these annotations can possibly provide information to pinpoint genomic regions with high functional potential. Under this setting, the logit link function can perform better than the probit link function as what we observed for the muscle skeletal tissue.

Q.1.5. The GWAS SNPs with active epigenetic annotation are prioritized. I wonder how they treated the GWAS SNPs without clear annotation.

R.1.5. When we built the gene expression imputation models, we set the missing annotation values as 0. Therefore, we would include all available cis-SNPs in the GTEx dataset when building gene expression imputation models, whether they have annotation information or not. In the following step of integrating GWAS SNPs to identify disease-associated genes, we remove those GWAS SNPs not included in the gene expression imputation models, as is what is commonly done in the widely used TWAS frameworks, e.g. prediXcan. Therefore, for GWAS SNPs without clear annotation, as long as they are included in the imputation models, we would also include them in the test.

Q.1.6. Bayes methods are known to be computational intensive, especially for high-dimensional genotype data. The authors used the variational Bayes method which is less computational intense compared to MCMC. I wonder if the authors can provide the length of hours for imputation of gene expression on each tissue type. I assume that it takes longer in some tissue types and shorter in other tissue types.

R.1.6. Thank you for raising the computational issue of our method. Compared with other optimization methods like elastic net models, Bayes methods are computationally more intensive. In the revised manuscript, we provide some statistics about the running time in the S1 text of the revised manuscript. As the imputation models only need to be trained once and can be applied to any GWAS data after training, we see the computation burden may be justified if the models can yield more replicable disease-gene associations.

“*The running time of our method is longer than that of elastic net (glmnet R package). For example, when fitting models on 100 sets of data with 100 individuals, 1,000 SNPs and 5 kinds of annotation array, our method takes 15.15 minutes while the elastic net takes 13.90 seconds on the same CPU (Intel(R) Xeon(R) CPU E5-2620_v3 2.40GHz).* *More specifically, the running time for model training in each tissue can be found in the S11 Table.*”

Reviewer #2: Liu et al. proposed an interesting idea to incorporate epigenetic annotation into transcriptome-wide association studies (TWAS). They adopted a Bayesian variable selection model to integrate Roadmap epigenetic annotations, GTEx data, and LD hub GWAS summary statistics. Through intensive analyses of 207 traits on LD hub, they demonstrated advantages of their proposed method T-GEN over other methods. Then they thoroughly discussed a detailed application to Alzheimer’s disease. Below I list some comments that may help improve the manuscript.

We thank the reviewer for careful reading of our manuscript and giving a succinct and positive summary. We also thank the reviewer for the detailed comments that are addressed below.

Q.2.1. It is worrisome that using GTEx brain cortex BA9 data to impute CommonMind Consortium (CMC) BA9 brain data can only achieve R2 of 0.007. Given GTEx data are only healthy controls, how about only considering CMC controls data with the same age range as GTEx subjects?

PrediXcan published its prediction model online (<http://predictdb.org/>), including for a newer version of GTEx data (V8). What is the R2 directly using their prediction weights?

It is also counterintuitive that improving gene expression imputation accuracy does not help TWAS. How many genes are left after the FDR filtering?

R.2.1. Thank you for your insightful comments. As noted, the GTEx data are only collected from presumably healthy controls. One essential step in all the TWAS approaches proposed in the literature, including T-GEN, is to accurately impute gene expression for individuals, including both normal and diseased individuals. Therefore, it is important for gene expression imputation models to have acceptable accuracy in both case samples and control individuals. Therefore, it would be more suitable to evaluate imputation models in datasets with both case and control samples, like the CommonMind data. Even though, for a fair evaluation of the trained imputation models in the GTEx dataset, we have evaluated the prediction performance on the control samples only. Compared with considering all samples, all models achieved significantly (all p<1e-10) better prediction $R^{2}$ (**elnt**: 7.2e-3 to 1.2e-2; **elnt.annot**: 7.2e-3 to 1.2e-2; **vb**: 7.3e-3 to 1.0e-2; **vb.annot**: 7.0e-3 to 1.1e-2; **T-GEN**: 7.1e-3 to 1.1e-2) on the normal samples.

We agree with the reviewer on the performance of the imputation models as a function of the available sample size. We applied the prediXcan models (v8) and mash (v8) models, both of which were downloaded from http://predictdb.org, to predict the gene expression levels for all the samples in CommonMind. Mash is a more recent method for estimating and identifying genetic effects (*Nature Genetics* 51, no. 1 (2019): 187-195). Similar to our results based on GTEx v6p, the prediction accuracy did not show significant improvement, PrediXcan (v8) models having $R^{2}$ of 0.0076 (elnt v6: 0.0072, p=0.09), mash (v8) models having $R^{2}$ of 0.0073 and T-GEN models (v8) having $R^{2}$ of 0.0070 (T-GEN v6: 0.0071, p=0.11). We also investigated the impact of the increased sample size on the prediction accuracy for normal samples only. Compared with elnt and T-GEN models trained on GTEx v6, GTEx v8 models did not show significantly (both p>0.2) better prediction performance on the control samples (**elnt**: 1.2e-2 to 1.1e-2; **T-GEN**: 1.1e-2 to 1.1e-2). We have added these results in the S1 Text.

We also agree with the reviewer that it is a complex problem to study the relationship between imputation accuracy and the power to identify disease-associated genes. This is now discussed in the supplementary text:

“*We further investigated the impact of imputation accuracy on the identification of disease associated genes. We did not observe a consistent pattern between imputation accuracy and the percentage of associated genes identified with pLI>0.99 across the five methods (****S7 Fig****). Similarly, the percentage of genes identified associated with 207 traits did not show a consistent pattern between imputation accuracy across five methods either (****S8 Fig****)*.”

After FDR adjustment, there were 262,348 significant tissue-gene models across 26 tissues. The number of significant imputation models for each tissue can be found in the “model number” table provided in the supplementary table.

Q.2.2. The authors claimed T-GEN discovered more genes. Is there any empirical check of the distribution of the p-values to ensure that there is no inflated FDR?

R.2.2. Thank you for raising the possibility of having different levels of false positives in identifying trait-associated genes across different methods. Because it is difficult to identify false positive findings of trait-associated genes purely based on p-values, we resorted to replicate our findings in AD using summary statistics from an independent AD GWAS: “*Among the 96 genes identified by our method using the GWAS of clinical AD, 50 (52%) genes were successfully replicated in this independent GWAS study (p < 5.2e-4) including TMEM135 in the novel loci. For the other four methods, only 9.9% (8/81, vb) to 41% (33/81, elnt.annot) genes were replicated in the GWAX data*.”

Q.2.3. The GTEx data have been updated to V8. Given the largely increased sample size, it may help boost gene expression imputation accuracy and association discovery if the authors can update the analyses to GTEx V8 data. If not for all the traits, updating the main application (Alzheimer’s disease) would show the impact of sample size.

R.2.3. Thank you for your suggestion and we do agree with the importance of updating the imputation models to GTEx v8. Due to the time limit on the revision, we have updated the imputation models in two tissues: the whole blood and liver tissue since these two tissues were found to be enriched for GWAS signals for AD (estimated by LD score regression, see the “AD enrichment” table in the supplementary table). We have also tried to evaluate the impact of sample size on gene expression imputation through the analysis of an external dataset and the genes identified to be associated with AD. Firstly, we used the elastic net models on GTEx v8 (trained by prediXcan) to predict gene expression of the CommonMind data and did not observe significant (p=0.09) prediction accuracy improvement (v6 $R^{2}$:0.0072, v8 $R^{2}$: 0.0076), see details in R.2.1. and the additional discussion in the S1 Text.

In terms of gene-trait association analysis, we found that increased sample sizes did not necessarily lead to more genes identified and we have added the results in the main text as

“*To further evaluate the impact of sample size on training gene expression imputation models, we built imputation models in AD heritability enriched tissues (liver and whole blood) using the GTEx v8 data and further identified AD-associated genes in these two tissues (****S9 Table****). Compared with using the GTEx v6 data, elastic net models by prediXcan (16 in v8 vs. 15 in v6) and T-GEN models (22 in v8 vs. 28 in v6) did not show substantial increase in the number of genes identified. However, with increased sample size in GTEx v8, we observed higher replication rates for the identified disease-associated genes using the GWAX data. For elastic net models, the replication rate increased from 17.6% to 66.7% while for T-GEN models, the rate increased from 20.7% to 75% in these two tissues.”*

Q.2.4. Fig 2 shows the percentage of functional SNPs. Can the authors show the number of selected SNPs by each method? Why two methods (elnt.annot and vb.annot) are not compared here?

The authors acknowledged that “We note that T-GEN utilizes the epigenetic information in SNP selections, and the same information is also used in ChromHMM models. Therefore, we expect to select more SNPs annotated as functionally active in ChromHMM models. To further evaluate the functional potential of the SNPs selected by T-GEN, we considered the CADD scores of the identified SNPs across all five methods.” So what presented in Fig 2 is an unfair comparison, and it seems to make more sense to demonstrate the results of CADD scores in Fig 2.

R.2.4. The number of selected SNPs by each method in each tissue is shown in **Figure S1**. Overall, T-GEN (a mean level of 45,084 SNP-gene pairs in a tissue) did not select significantly more SNPs than the other methods (elnt: 143,358) while the identified SNPs are more enriched with functional potentials.

In elnt.annot and vb.annot, we only used SNPs with H3K4me3, H3K4me1, and H3K9ac signals to build gene expression imputation models. These three epigenetic marks are highly associated with functional potential and were used in ChromHMM models for functional annotations. Therefore, elnt.annot and vb.annot would naturally identify a much higher percentage of functional SNPs. Therefore, it is difficult to consider these two models in comparisons. We have added comparison results in **Table** **S2**. As expected, vb.annot and elnt.annot showed the highest ratio of SNPs with CADD scores larger 20. We have combined the CADD score results with previous figures in the new Figure 2.

Q.2.5. Fig 3 is not cited in the paper. There are 8 main figures and 8 supplementary figures. The authors may consider combining similar figures into a bigger figure or compile all supplementary materials in a single file. Now it takes time to download and review supplementary figures one by one.

R.2.5. Thank you for pointing out this oversight in our initial submission. We have updated the citation of original Figure 3 after combining similar figures into one more comprehensive figure in the revised manuscript as

“*With the same FDR cutoff, T-GEN had an increase in the range of 2.8% to 55.3% (3,807 in whole blood) for the number of gene expression imputation models in each tissue, compared with the elastic net methods in 25 of the 26 tissues (****Fig 2C, D****)*.”

We have also compiled all supplementary materials into one single file.

Q.2.6. As a computational biology paper, would the authors provide analysis code or software?

R.2.5. Yes, the code for building gene expression imputation models and also gene-trait association test will be made publicly available once the manuscript is accepted.

Q.2.7. The authors considered 26 tissue types with Roadmap and GTEx data. For instance, for the application to Alzheimer’s disease, they identified loci in tissue types like ovary and lung. I am not sure if this makes sense in biology. The authors may pursue further on trait-relevant tissue types. E.g., 1) provide heritability enrichment for each identified tissue type; 2) weight tissue types by trait relevance in the testing; 3) only consider relevant tissue types by a cutoff of heritability enrichment. This would also help release the burden of multiple testing.

R.2.7. We thank the reviewer for this suggestion. Due to limited sample sizes in certain tissues like brain tissues, we caution that the TWAS frameworks including T-GEN here may have reduced power in identifying trait-associated genes if we only focus on trait-associated tissues.

Using LDSC (*Nature genetics* 47, no. 11 (2015): 1236.) and annotations from GenoSkyline-Plus (*PLoS genetics* 12, no. 4 (2016): e1005947.), we found that AD showed significant heritability enrichment in immune tissues, which is similar to whole blood considered here (4.05-fold, p=1.34e-4, using annotation Tier 2) and also in liver (10.9 fold, p=1.96e-5, Tier 3). As a result, we have focused on these two AD-related tissues to identify associated genes and the results are shown in the last part of R.2.3.

Typos:

1. “gens” appeared four times, including when mentioning the full name of the proposed method T-GEN. Should it be “genes”?

2. “relavant” in the caption of Fig S2.

3. S1 Text mentioned that there are 208 traits other than 207 stated in the main text.

Thank you for pointing out these typos. We have corrected them in the main text and also S1 Text.

Reviewer #3: The main idea of this study is to add epigenetic information in gene expression imputation from eQTL SNPs and this helps to identify more statistically predictive and biologically functional SNPs, which leads to the identification of more trait-associated genes. In the process of imputing expression with SNP information, the epigenetics annotation was used to set priorities among candidate SNPs. Experimental results show that the proposed method can select more potent factors than the previous methods.

We thank the reviewer of summarizing the main idea of our manuscript and we have addressed the comments as follows:

Major comment:

Q.3.1. The study is based on the assumption that SNPs with active epigenetic annotation are more likely to modulate tissue-specific gene expression. Reference to support this or experimental validation would be necessary (paragraph 2 in Introduction).

R.3.1. We thank the reviewer for raising this important issue. We have added the following text and reference papers as

“*To more accurately identify regulatory eQTLs that play functional roles, we assume that SNPs with active epigenetic annotations are more likely to regulate tissue-specific gene expression [14–18].”*

14.	Geyer PK, Green MM, Corces VG. Tissue-specific transcriptional enhancers may act in trans on the gene located in the homologous chromosome: the molecular basis of transvection in Drosophila. EMBO J. 1990;9: 2247–2256. Available: https://pubmed.ncbi.nlm.nih.gov/2162766

15.	Ong C-T, Corces VG. Enhancer function: new insights into the regulation of tissue-specific gene expression. Nat Rev Genet. 2011;12: 283–293. doi:10.1038/nrg2957

16.	Bell JT, Pai AA, Pickrell JK, Gaffney DJ, Pique-Regi R, Degner JF, et al. DNA methylation patterns associate with genetic and gene expression variation in HapMap cell lines. Genome Biol. 2011;12: R10. doi:10.1186/gb-2011-12-1-r10

17.	Swift-Scanlan T, Smith CT, Bardowell SA, Boettiger CA. Comprehensive interrogation of CpG island methylation in the gene encoding COMT, a key estrogen and catecholamine regulator. BMC Med Genomics. 2014;7: 5. doi:10.1186/1755-8794-7-5

18.	Kumar D, Puan KJ, Andiappan AK, Lee B, Westerlaken GHA, Haase D, et al. A functional SNP associated with atopic dermatitis controls cell type-specific methylation of the VSTM1 gene locus. Genome Med. 2017;9: 18. doi:10.1186/s13073-017-0404-6

Q.3.2. As the authors noted, it is naturally expected to select more SNPs annotated as functional by adding epigenetic information in the imputation process. Therefore, further validation of selected SNPs is done by showing that the T-GEN identified eQTLs have higher CADD scores, while the score improvement is marginal. The final validation is done through the identified train-associated genes, which are more in number and include higher percentage of functionally conserved genes. Still, it doesn’t seem to fully validate the potential advantage of the proposed method. Since the main idea in Methodological perspective is to add epigenetic annotation information, it would be helpful to do some experiments regarding the robustness against the bias or incompleteness of the annotation information. Simulation study on this and other aspects that can show the performance behavior of the proposed method would be useful.

R.3.2. We agree with the reviewer that incompleteness of the annotation information can have impact on model performance. To alleviate the potential impact of limited sample sizes, we focused on the muscle skeletal tissue in GTEx v8 to assess the impact of incomplete annotations. We have added the results in the S1 Text as follows:

“*By considering different proportions (5%, 15%, 25%) of SNPs with missing annotations, where we set the missing annotation values as 0, we further evaluated the impact of incompleteness of annotations. Different levels of incompleteness did not show significant impacts on imputation accuracy (****S9b Figure****) though models built with the full annotation information achieved the best accuracy (*$R^{2}$*) of 0.075 (5% missing: 0.07, 15% missing: 0.07, 25% missing: 0.07). As the missing rate increased, the fraction of SNPs with CADD scores larger than 20 decreased (original: 0.69%; 5% missing: 0.64%; 15% missing: 0.57%; 25% missing: 0.53%). Only when compared with models with the highest proportion of missing annotation 25%, T-GEN with full annotation information showed a statistically significant difference (p=4e-3) in identifying SNPs with CADD scores > 20. Overall, annotation missingness may affect T-GEN performance with moderate level of missingness.*”

This is consistent with our expectation that we will see reduced performance with incomplete annotation information.

Q.3.3. This method was compared to Elnt and vb, which are relatively classic methods. I’m

wondering if there is more recent research or method to use for comparison.

R.3.3. We agree with the reviewer that elnt and vb are relatively classic methods, therefore in the case study of late onset Alzheimer’s disease (AD), we have added comparison results with another new method named mash. Since the mash models were trained using the GTEx v8 data and due to high computation burden of T-GEN, we focused on AD heritability-enriched tissues, liver and whole blood in our comparisons. We have added the following text on page 13 of the main text:

“*We also compared the performance of T-GEN models trained in GTEx v8 with a recent method named mash (Nature genetics 51, no. 1 (2019): 187-195). Compared with the most updated models built on GTEx v8 for prediXcan (trained using elastic net) and mash, T-GEN identified the largest number of genes (22 vs. 18 by mash and 16 by prediXcan) and maintained a high replication rate in the GWAX data (75% vs. 76.2% in mash and 66.7% in prediXcan).”*

We also compared the imputation accuracy of mash with that of T-GEN models, and have added the results in the S1 Text as follows:

“*The prediction accuracy did not show significant improvement with prediXcan (v8) models having* $R^{2}$ *of 0.0076 (elnt v6: 0.0072, p=0.09), mash (v8) models having* $R^{2}$ *of 0.0073 and* *T-GEN models having* $R^{2}$ *of 0.0070 (T-GEN v6: 0.0071, p=0.11).*”

Q.3.4. In the section “More genes can be effectively imputed by T-GEN”, more detailed explanation of the measurement method is needed. In addition, there should be comparison of prediction errors as to whether the proposed method predicts expression matrix Y well from X.

R.3.4. We have added the following statement “*For gene-trait association identification, only gene expression imputation models with significant squared correlation between the observed and imputed expression levels (*$R^{2}$*) (FDR < 0.05) were considered*” in the Model training and evaluation part of the method section.

As for prediction errors, we have included the imputation accuracy evaluation in the external dataset CommonMind and also the five-fold cross validation accuracy evaluation in the S1 Text. Also, in the revised version, we have updated the prediction results in the CommonMind dataset using the updated T-GEN v8 models, see R.3.3.

Q.3.5. Figure 3 is not explained in the main text. Also, there is an elnt category in Figure 3, and it

seems that the values are all zero. If it is just a control (all 0), it may be deleted.

R.3.5. We thank the reviewer for pointing this out. We have updated the citation of the original Figure 3 and updated the figure by removing the “elnt” category as

“*With the same FDR cutoff, T-GEN had an increase in the range of 2.8% to 55.3% (3,807 in whole blood) compared with the elastic net methods in 25 of the 26 tissues (****Fig 2C, D****)*.”

Typo:

In line 297, “gens” should be “genes”.

We thank the reviewer for pointing out the typo. We have corrected it in the main text and also S1 Text.
