# Supplementary material for: Leveraging functional annotation to identify genes associated with complex diseases
Source: PLoS Comput Biol. 2020 Nov 2;16(11):e1008315. doi: 10.1371/journal.pcbi.1008315 (PMC7660930; doi:10.1371/journal.pcbi.1008315)
Supplement: Attachment — Submitted filename: reviewer_comments_final.docx [file pcbi.1008315.s025.docx]

**We thank all the reviewers for their valuable comments. We have revised our manuscript to address these comments as detailed below.**

**Reviewer #1:** I believe the authors have appropriately addressed my comments. I feel that the authors used many 'can' in sentences. Some of the 'can' should be changed to 'may', other 'can' should be removed and just use the verb. For example, “H3K9ac [can] be present in both actively transcribed and bivalent regions." In this sentence, 'can' should be 'might'. Another example, "More specifically, the running
time for model training in each tissue [can] be found in the S11 Table", here should be ".... in each tissue was displayed in the S11 Table". Another "the annotation configuration and
incompleteness [can] also affect the results", here "can" should be "may", "More genes [can] be effectively imputed by T-GEN", here "can be" should be changed to "are"

We thank the reviewer for his/her comments. We have changed some of the ‘can’s used in the manuscript and the supplementary text. Besides what was suggested by the reviewer, we have also changed some other can’s. For example,

1. Line 63, “*T-GEN not only can identify eQTLs with higher…*” to “*T-GEN not only identified eQTL…*”.
2. Line 155, “*In general, we can describe the relationships…*” to “*In general, we describe the relationships…*”.
3. Line 177, “*Overall, these results demonstrate that our method can better identify…*” to “*Overall, these results demonstrate that our method better identified…*”.

More edits can be found in the main text and also the supplementary text.

**Reviewer #2:** My comments have been addressed.

We thank the reviewer for taking the time to review our manuscript.

**Reviewer #3:** This paper proposes a new method of using functional annotation to identify disease-related genes and it seems a significant contribution to the field. Most of the concerns I have raised about the previous version have been resolved and I recommend to accept this manuscript after minor edits, e.g.
- line 336: substantial number increase in the number of genes --> substantial increase in the number of genes

And the authors would need to include in the manuscript a link that provides the used data and software/code.

We thank the reviewer for his/her comments. We have edited line 336 (now line 349) to “*did not show substantial increase in the number of genes identified*”.

The software and the pre-trained models can be found at <https://github.com/vivid-/T-GEN>. We have also added this on the line 162 of the main text “*The implemented method and pre-trained models are available on https://github.com/vivid-/T-GEN*”.
